# A simple survey protocol for assessing terrestrial biodiversity in a broad range of ecosystems

**DOI:** 10.1371/journal.pone.0208535

**Published:** 2018-12-12

**Authors:** Asko Lõhmus, Piret Lõhmus, Kadri Runnel

**Affiliations:** 1 Department of Zoology, Institute of Ecology and Earth Sciences, University of Tartu, Vanemuise, Tartu, Estonia; 2 Department of Botany, Institute of Ecology and Earth Sciences, University of Tartu, Lai, Tartu, Estonia; University of Sydney, AUSTRALIA

## Abstract

Finding standard cost-effective methods for monitoring biodiversity is challenging due to trade-offs between survey costs (including expertise), specificity, and range of applicability. These trade-offs cause a lack of comparability among datasets collected by ecologists and conservationists, which is most regrettable in taxonomically demanding work on megadiverse inconspicuous taxon groups. We have developed a site-scale survey method for diverse sessile land organisms, which can be analyzed over multiple scales and linked with ecological insights and management. The core idea is that field experts can effectively allocate observation effort when the time, area, and priority sequence of tasks are fixed. We present the protocol, explain its specifications (taxon group; expert qualification; plot size; effort) and applications based on >800 original surveys of four taxon groups; and we analyze its effectiveness using data on polypores in hemiboreal and tropical forests. We demonstrate consistent effort-species richness curves and among-survey variation in contrasting ecosystems, and high effectiveness compared with casual observations both at local and regional scales. Bias related to observer experience appeared negligible compared with typical assemblage variation. Being flexible in terms of sampling design, the method has enabled us to compile data from various projects to assess conservation status and habitat requirements of most species (specifically rarities and including discovery of new species); also, when linked with site descriptions, to complete environmental assessments and select indicator species for management. We conclude that simple rules can significantly improve expert-based biodiversity surveys. Ideally, define (i) a common plot size that addresses multiple taxon groups and management goals; (ii) taxon groups based on field expertise and feasible number of species; (iii) sufficient and practical search time; (iv) a procedure for recording within-plot heterogeneity. Such a framework, combined with freedom to allocate effort on-site, helps utilizing full expertise of observers without losing technical rigor.

## Introduction

Lack of data on species-rich inconspicuous land organisms remains a major knowledge problem in addressing the challenge of global biodiversity loss. For example, monitoring of the status of terrestrial biodiversity is mainly based on vertebrates [1] that form 2% of known species, while over 80% of terrestrial species are still undescribed [2]. For land use planning at local and regional scales, research has identified many ‘indicator taxa’ but few are actually used [3] and these usually represent conspicuous organisms of direct human value, such as trees, game animals, or wild berries [4]. Even in Europe, that has long biodiversity research traditions, some major functionally important groups remain almost ignored (e.g., soil organisms) [5], and the distribution, trends, and key pressures on uncommon taxa and full species diversity are only fragmentarily known [6]. In terms of management, monitoring of threatened species remains the clearest gap in addressing, for example, sustainability of forest management [7] or conservation of otherwise well-known taxon groups [8]. Although these problems are obvious to researchers, they have turned out to be fundamental enough to resist solution.

The problems with surveying megadiverse taxon groups including, specifically, rare and threatened species, emerge from two main sources (see also [9]). First, field survey and morphological identification of most taxa requires specific expertise, which is limited overall and further scattered between research disciplines (notably taxonomy, biogeography, ecology, conservation). To overcome this limitation, field identification can be replaced with collecting all specimens for lab inspection [e.g., 10,11] but it can raise ethical concerns, and is obviously problematic at large scales, for repeating the surveys, or studying rare and threatened species. There is some hope in a recent spread of taxonomic expertise and field research to amateur level [12,13], but wide coverage of biodiversity is still rare in environmental decision-making. Secondly, the survey methods vary significantly among experts and disciplines, leading to scarcity of standard data for cross-disciplinary syntheses and for management purposes (estimating biodiversity trends; comparing sites or the consequences of alternative actions; etc.). For example, taxonomists typically sample casually over wide regions and in their favourite habitats [14], and such biased data are of limited use for other purposes [6,15]. Ecological studies and environmental monitoring schemes use well standardized survey methods and sampling designs [16], but may lack conservation relevance either due to poor coverage of rare species [17,18] or different spatial scales [19]. The specific sampling designs for rare species [20] are not generally cost-effective for wider biodiversity monitoring. Several pitfalls and challenges also limit the inclusion of rapidly evolving methodologies of mass DNA- sampling into the conservationists’ toolkit [21,22].

It follows that one way to enhance biodiversity surveys is to optimize the expert labour – by developing robust survey methods that would allow a species expert to simultaneously address rewarding ecological, taxonomic, and conservation topics. Unexpectedly, this line of thought remains rare. Hunter and Webb [9] pioneered in outlining a recommendation to field lichenologists – assemblage-level surveys that follow ecological designs of conservation relevance (plots >2 ha in size; standard efforts; basic environmental descriptions of the plots). Their flexible approach did not, yet, scrutinize a field protocol, address survey repeatability or demonstrate its advantages based on a realized scheme. Most other approaches, however, have focussed on fewer joint benefits. For example, rapid field assessment programs, as applied by conservation organizations worldwide, consolidate taxonomists and conservationists to record new or rare species in remote places, but their methodological looseness limit ecological (statistical) analyses and longer-term conservation perspectives [23]. An opposite is exemplified by many biodiversity monitoring programs with small plots randomized or systematically placed over areas of interest – working through a mass of common situations and species is neither attractive for taxonomists nor effective for discovering species or sites of conservation concern. In standard arthropod trapping (e.g., Malaise trap programs), the mass material collected may simply exceed taxonomists’ handling capability in practical conservation time-frames (but see [24,25] for DNA barcoding perspectives). Conservation relevance is also unclear in general calls to ecologists to include diverse taxon groups in their studies – with the help from professional taxonomists who could gain from high-profile publishing and improved funding [26].

In this paper, we present and analyze a simple field protocol for standard surveys of a broad range of macroscopic sessile (or only locally mobile) land organisms, which can produce data for diverse questions in conservation, ecology, and taxonomy. The protocol has been built on many principles described in [9] but we have re-considered goal-setting and practical trade-offs in order to allocate expert’s field effort to simple priority tasks. Our main theoretical consideration includes links between (i) non-random distribution of species within ecosystems, and (ii) the special training of field taxonomists to rapidly detect, within their group of expertise, both specimens likely to represent distinct taxa and their main microhabitats present at a given site. In such case, we expect that (iii) *taxonomists can compile raw species lists in the field most efficiently when they move around*, *guided by their expertise and diminishing returns of new information* (like time-limited ‘optimal foragers’; e.g., [27]). Research on human visual searches supports such optimal switching behaviour even when the number of objects is unknown [28]. A key factor for survey quality is a sufficient effort, which can be identified either from effort-species richness curves or pre-specified detection probabilities of individuals or species. Because we expect taxonomists to discover and collect species in any area more efficiently than when using pre-defined subsampling, *the area can be delineated according to ecological and conservation goals*. For our protocol, such broad goals include: repeatability in time and applicability in contrasting ecosystems and for any plot arrangement; species abundance and habitat data that can be used in formal red-listing procedures [29]; quality data for species habitat and distribution modelling (including absences; cf., [30]); and analyses of assemblage composition and structure (including cross-taxon and bioindicator assessments).

The paper is organized as follows. First, we present a formal survey protocol and list the datasets collected following this protocol on polypore fungi, lichens, bryophytes, and vascular plants. We term the general approach ‘*fixed-area-fixed-effort survey*’ to emphasize its critical requirements and to distinguish it from any species expert-based mesoscale approach (termed ‘floristic habitat sampling’ by [18]). Secondly, we explain the specifications of our protocol: target taxa and ecosystems; expert qualification (including the learning effect); the fixed plot size and survey effort used (4 hr search on 2-ha plot). Thirdly, we use our original datasets on polypore fungi to analyze the effectiveness of the survey method for describing local species pools. These mostly wood-inhabiting fungi form an ecological group with typically a few hundred species present in regional species pools; they have been popular targets in assessments of forest biodiversity and management impacts [31,32]. Finally, we provide an overview of the data analysis opportunities and the potential for management insights.

## Methods

### A field protocol of the fixed-area-fixed-effort survey

**Fig 1:**
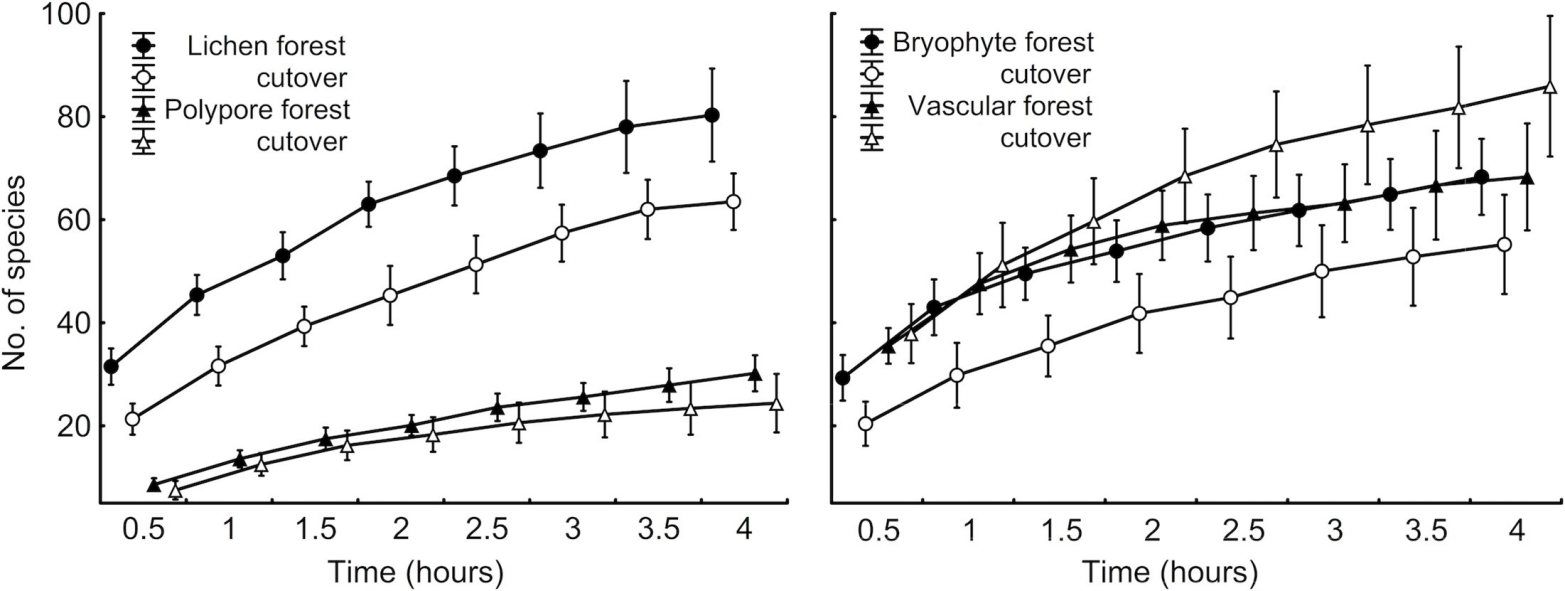
Mean species accumulation of four taxon groups by 0.5-h survey intervals in Estonian swamp forests. Data for the 2-ha plots in 10 old stands and 10 post-clearcut stands are shown separately; the whiskers are 95% CI. Note the overlapping curves of vascular plants and bryophytes in forests, but their opposite responses to clear-cutting (right).

1. **Specify the target of the individual survey or survey program in terms of the taxon group and statistical population** (e.g., lichens in a particular reserve or in certain types of forest in a region). Usefulness of a single survey is limited to remarkable records, thus comparative approaches containing multiple plots (categorized or along gradients) or target plots against reference plots are recommended.

2. **Delineate a 2-hectare plot to represent the land cover of interest.** Prefer compact shapes (e.g., rectangular plots), avoid disjunctions that restrict observer movement among parts of the plot. Plan borders that can be followed in the field. Be explicit about: what is considered within-plot heterogeneity (e.g., forest gaps, rocks, small depressions) and what should be excluded as ecologically different land cover (e.g., roads, watercourses); how are sharp land-cover edges addressed (be consistent in either avoiding or including these). Replicate plots whenever possible, using established sampling designs (stratified random or block designs often preferable for ecological analyses).

3. **Establish plot borders in the field in a way that they can be (i) tracked during the survey with minimum loss of attention, and (ii) repeated during re-surveys.** A combination of accurate maps, marking of corners in the field, and using GPS device is usually sufficient. Record plot borders in a GIS as metadata for analyses of broad biodiversity patterns.

4. **Minimize the number of observers (species experts) carrying out the surveys in different plots.** Because the survey method is based on individual decisions in the field, it is best to use the same observer throughout a program. If that is not possible, start with joint training surveys to standardize the field procedures and, in the actual program, allocate the variation among plots equally between persons. Prefer established experts capable of keeping high survey speed for hours.

5. **Plan for a 4-hour survey in good weather conditions, preferably during a single day.** The duration can be slightly shortened in very poor and extended in very rich assemblages based on the accumulation of new records (see below). Crucial features are the survey speed, and the observer’s memory of locations and microhabitats already checked and the species or specimens recorded. Adverse conditions decreasing the survey speed should be minimized, e.g., rainy conditions that also obstruct making notes and collecting. Splitting surveys between two subsequent days is acceptable as the observer usually remembers the situation, but longer intervals between the surveys are not recommended. For seasonal organisms, longer intervals may even represent different assemblages.

6. **Standardize field season (and year).** In temperate and boreal areas, bryophytes and lichens can be surveyed during whole snow-free seasons, although in deciduous forests fallen leaves can reduce ground-level detectability and frosts can affect field identificability. For seasonally appearing taxa, restrict surveys to top detectability seasons or repeat surveys consistently over seasons. Yearly fluctuating occurrences (such as fruit-bodies of most fungi) should be sampled according to the survey goals – either by restricting all surveys to a single year, repeatedly sampling over multiple years, or distributing single visits among years.

7. **Keep the priority order of tasks throughout the survey: (i) record all species in the plot and collect at least difficult-to-identify specimens; (ii) assess abundance of each species, including individual records of rare species; (iii) list microhabitats of each species.** Such order of tasks determines the observer’s decisions of switching locations and the time expenditure on taking notes and collecting specimens. Identify species rapidly in the field; if that is not possible, collect specimens considering the order of tasks (i.e., prioritize potentially unrecorded species; see also [33]). Collect operatively (packages prepared beforehand; all difficult specimens from one substrate into one package; simple number labels; etc.). Collecting fewer than ca. 35–40 samples per plot does not affect the survey speed much; if more are expected in diverse or poorly known assemblages, consider consistent using of a field assistant for collecting the specimens. Minimize other activities (photographing; collecting for museum; etc.) during the survey and, when they are undertaken, stop the survey time count.

8. **List species and individuals in the finding sequence, and distinguish time intervals for the records.** This enables analyses based on record accumulation over time (we recommend 0.5-hour intervals, see Fig 1) and, potentially, for comparing surveys of different duration. For field-layer plants and cryptogams record functional individuals and be explicit about the concept used. We recommend the concepts used for red-listing species, e.g. [34,35] for bryophytes and macrofungi (including lichens). Similar rationale in field-layer vascular plants can distinguish singular ‘individuals’ of non-clonal species; distinct small ‘clumps’ or clones (up to ca. 1 m^2^ size); and patches of near-continuous cover (‘local dominance’ for large patches of >1% of the 2-ha plot). Distinguish species that are only observed as dead, since the current environment may be unsuitable for them.

9. **Use the knowledge acquired during the survey to adjust moving between locations and accomplishing the three tasks (p. 7).** Minimize differences in prior knowledge on survey plots. When starting the survey (time count), first list all species you see at that point, and then start moving around by adding new species and their abundance and microhabitat data. Score the abundances of frequent species as soon as possible, to minimize time on counting their individuals (p. 10). Visit distinct microhabitats to find new species and improve the microhabitat lists for species already found. Avoid double counting, for example, by GPS tracking or leaving marks on locations visited. Avoid procedures that might, in terms of time expenditure, be incomparable among persons (e.g., climbing to trees); be explicit about tools improving detectability (e.g., binoculars for scanning tree trunks beyond reach). At the end of the survey, specify whether some parts of the plot remained poorly surveyed.

10. **Use an ordinal abundance scoring system (5–10 classes) with higher resolution at the rare end (e.g., score 1 for a single individual).** Such system prioritizes species list over abundance estimation (p. 7), explicitly documents rarities of taxonomic or conservation interest, and highlights potential outliers (e.g., singletons) for ecological analyses. Whenever feasible, we recommend to use the concept of ‘individual’ as recommended by the IUCN red-listing procedures (e.g., [34,35]). Count 10–15 individuals per species explicitly (can be rescored by necessity); for frequent species, use at least two abundance classes to distinguish sparse and dominant species (where feasible, provide also the approximate number of individuals actually seen).

11. **List microhabitats for each species using consistent, pre-defined categories.** A microhabitat list improves re-scalability of the records and enables to analyze within-plot heterogeneity (particularly when combined with detailed plot descriptions, p. 12). The list should be straightforward (cf. p. 7) and informative both about species (natural history) and their microhabitats (assemblages). Two convenient alternatives to a general qualitative list are: (i) listing records on selected microhabitats, e.g., field-layer vascular plants on fallen logs, wet depressions, etc.; (ii) recording details (e.g., frequency distribution or measurements) in a haphazard sample of first records (e.g., [36]).

12. **Separately from the species survey, describe the environment of the plot using standard ecological methods.** Consider study goals, taxon groups, repeatability, and management relevance. For up-scalability, we also recommend recording the plot context, at least at level of surrounding land cover, and ecosystem-change driver values according to established systems (e.g., [37]).

### Datasets and analyses

In 2005–2017, our team has used the protocol for >850 surveys in four European and two South American lowland countries (Table 1). In the current paper, we use polypores as the main test group for illustrating the method, but we also present selected results on three other groups studied in the same experimental designs for a comparison (data in S2 Table). Our surveys have focused on forest landscapes (including open early-successional areas) and the human influence on these ecosystems (comparing, for instance, harvesting systems; tree planting with natural regeneration; and artificial drainage with natural development of wetlands).

**Table 1:** Overview of the material collected using the fixed-area-fixed-effort survey method in Europe and South America: taxon groups, field designs, and published reports.

Dataset^a^^,^^b^	Country	Field design	*N*^c^	Reports
**P1; L;** V; **B**	Estonia	Management stages of forests (block design)	440 (92)	[33,36–43]
**P2;** L; V; **B**	Estonia	High conservation value forests	84 (20)	[36,44]
**P3** L; V	Estonia	Artificially drained forested peatlands	114 (29)	[43,45]
**P4**; V	Estonia	Repeated survey of post-logging succession	81 (43)	[42]
**P5**	Estonia	Management stages of alvar forests (block design)	18 (18)	
**P6**	Estonia	Young eutrophic and pine forests	12 (12)	
**P7**	French Guiana	Intact vs. disturbed rainforests	9	
**P8**	Brazil	Intact rainforests vs. reclamation sites	14	
**B**	Estonia	Shelterwood harvested conifer forests	20	[46]
**L**	Estonia, Finland	Post-fire successional stages in pine forest	18	[47]
**L**	Finland, Lithuania, Belarus	Management stages of pine forest (block design)	37	[48]
L	Lithuania	Management stages of nemoral forest (block design)	12	
Total			**859 (237)**	

^a^ P1–P8, polypore datasets; B, bryophytes; L, lichens; V, vascular plants

^b^ datasets included in the observer experience analyses are in **Bold**

^c^ total no. of surveys across all taxon groups (no. of polypore surveys in brackets)

No specific permissions were required for surveys in state-owned non-protected sites in Estonia and Finland, and in the areas visited in French Guiana. Permissions to work in the Estonian strict reserves since 2005 were issued by relevant county environmental services and, after their reorganization in 2009, by the Estonian Environmental Board (the central body under the governance of the Ministry of the Environment). The collecting did not target endangered or protected species; however, the University of Tartu holds the permission, for research purposes, to remove (non-vital) parts of protected lichens and fungi. Permissions (to PL and AL) to survey in Finnish study sites in protected areas in Lieksa and Ilomantsi in 2010 were provided by Metsähallitus, Natural Heritage Services. In Brazil, the working permissions were provided to the Norwegian Centre for International Cooperation in Education (CAPES-SIU-2013/10057); the collecting permit was issued by ICMBIO to Adriene Soares.

To illustrate *protocol specifications*, we present two analyses based on multi-taxon datasets.

1. We illustrate the general rationale for a standard protocol to describe plant and fungal assemblages based on surveys in Estonian swamp forests, separating closed mature (>60 years) and open early-successional (2–9 years post clear-cut) stands (datasets described in [36,41]. We present mean species accumulation curves for lichens, polypore fungi, bryophytes and vascular plants by half-hour intervals as recorded following the protocol.

2. To measure potentially enhanced species discovery along with growing survey experience, we related the relative species richness recorded to the survey sequence in each of the three authors. We considered all the polypore surveys carried out by A.L. and K.R. (both these authors had only one year prior experience before this program), and lichen surveys and bryophyte surveys by P.L. who started as an experienced lichenologist but inexperienced bryologist. We (i) assigned each survey its order in the sequence and the habitat type (combination of forest type and successional stage; [36]); (ii) applied general linear models (STATISTICA 8 software; StatSoft; Tulsa, Okla, USA) that related the habitat type to the number of species recorded (dependent variable), and calculated the model residuals; (iii) regressed these residuals (i.e., relative species richness) against the survey order.

We address *survey effectiveness* to describe four dimensions of biodiversity in analyses based on polypore datasets (S1 Table). The datasets P1-P3 (Table 1; *N* = 143 study plots) represent five forest types in contiguous lowland forests in Estonia, in hemiboreal Europe (see [36, 43] for details). The forest types are defined by combinations of soil fertility and moisture conditions that host different tree assemblages either dominated by Scots pine (*Pinus sylvestris*) or mixtures of Norway spruce (*Picea abies*) and deciduous trees. In the old-growth stage, tree canopies in these forests typically reach 20–35 m height and live tree volumes range from 272±78 (SD) m^3^/ha in swamps to 473±135 m^3^/ha in meso-eutrophic mixedwood; coarse woody debris (CWD) volumes range from 14±12 m^3^/ha in dry pine forests to 198±45 m^3^/ha in moist eutrophic sites [49]. The two neotropical datasets, P7–P8, represent *terra firme* forests in neotropical moist forest zone. We analyze the data from 17 plots (8 in French Guiana, 9 in Brazil; Table 1). These forests are characterized by very species-rich tree layer with emergent trees reaching 45 m height [50]; the CWD stocks in old-growth stands have been reported to range from 11 to 31 tonnes ha^-1^ and live-tree stocks approximately ten times larger [51].

3. We illustrate applicability of the survey method in contrasting forest habitats (S1 Fig) by comparing mean polypore species accumulation curves: (i) in different biomes – old-growth stands in European hemiboreal vs. neotropical forests [datasets P1-P2 and P7-P8]; (ii) for logging impact assessment – mature production stands (66–79 years old at the time of the survey) vs. open clearcut stands (2–8 years post logging) of the most species rich Estonian forest type, eutrophic mixedwood [P1–P2]; and (iii) along natural gradients – compositionally similar pine stands on drained peat vs. sandy Podzol sites [P3; P1].

4. To assess the completeness of describing species pools, we performed two analyses using EstimateS [52] for extracting sample (survey) based rarefaction curves. The first analysis addressed how series of polypore surveys all over Estonian mainland [P1–4] described the regional species pool as listed after 50 years of thorough work by E. Parmasto [53]. We discarded the species only known from distinct sites not sampled in our program: anthropogenic habitats, alvar forests, and the Estonian western archipelago. The second analysis assessed the local species pool in two well-studied old-growth forests in Järvselja, eastern Estonia, where we have carried out multiple repeated inventories [P1, P4]. Since this site has also been well studied using casual surveys since the 1930s, we were also able to compare local effectiveness of traditional mycological surveys [54] and our rapid survey protocol.

5. We analysed the effectiveness of detecting a particular rare species based on *Rigidoporus crocatus* – a perennial resupinate polypore with average detectability [55] and of high conservation concern in northern Europe (e.g., [56]). For this species, we have recorded both exact time of detection since the start of the survey and the number of records; their relationship reveals the probability of finding the species depending on its local abundance.

6. Based on the data from polypore surveys in tropical French Guiana [P7], we illustrate the effectiveness to reveal assemblage differences in composition. The data matrix comprised 48 species (singletons removed) and was based on abundance scores (1–5). We visualized the assemblages using non-metric dimensional scaling with PC-ORD 6.07 package [57]; the procedure follows [36].

## Protocol specifications and explanations

The main specifications of the protocol are: the taxon groups and ecosystem types that can be surveyed (hereafter: ‘target groups’ and ‘target ecosystems’, respectively); the observer qualification needed; the plot area; and the survey effort.

**1. Target group.** The protocol applies to the taxon groups and situations where (i) a human observer can effectively discover individuals, i.e., sessile or low-mobility organisms or elusive taxa that reveal themselves by escaping behaviour, and (ii) identify most of the individuals to at least morphospecies level in the field; (iii) many species co-occur at the 2-ha scale in diverse non-randomly distributed assemblages. These features increase the merits of trained field taxonomists who (instead of random sampling) use their expertise for searching the habitat for all species and only need to collect few specimens for lab inspection. We have used the protocol mostly for plants and macrofungi (Table 1), but it can also be used for some animals. We have surveyed amphibians and reptiles; however, because these animals occurred sparsely in Estonia to meet the effectiveness criterion of species-rich assemblages (iii above), it was more cost-efficient to record them explicitly during vegetation surveys [58]. Others [59] have reported that freely moving observers detect more species of butterflies than when following strict transects of the same total area and time (10 min per 0.2 ha used in their study).

The target group must be explicitly defined [9] but, for comparing datasets and building up multi-taxon programs (Fig 1), we encourage further standardization based on taxonomists’ training and the broad environment. Focused surveys apparently capture target species more completely than when those species form a subset. Another aspect is the taxonomic knowledge needed. Top taxonomists can work with thousands of species (e.g., most macrofungi), but for wider applicability it is practical to divide such exceptional expertise between distinct surveys of smaller standard sets. We have used target groups (Table 1) that include 250–1500 species in regional species pools and perceived highest information gains when ca. 30–150 species are discovered during the 4-hour survey. In diverse assemblages, the time for taking notes becomes limiting (our top records, >220 species in vascular plants, refer to one species discovered every 1.5 min). Large species pools would thus require reduction of record detail or extra time. For example, in a broad-scale survey of lichens in burned forests, we did not include epilithic microlichens – a distinct, diverse and poorly collectable group that only occurs in certain regions [47].

**Fig 2:**
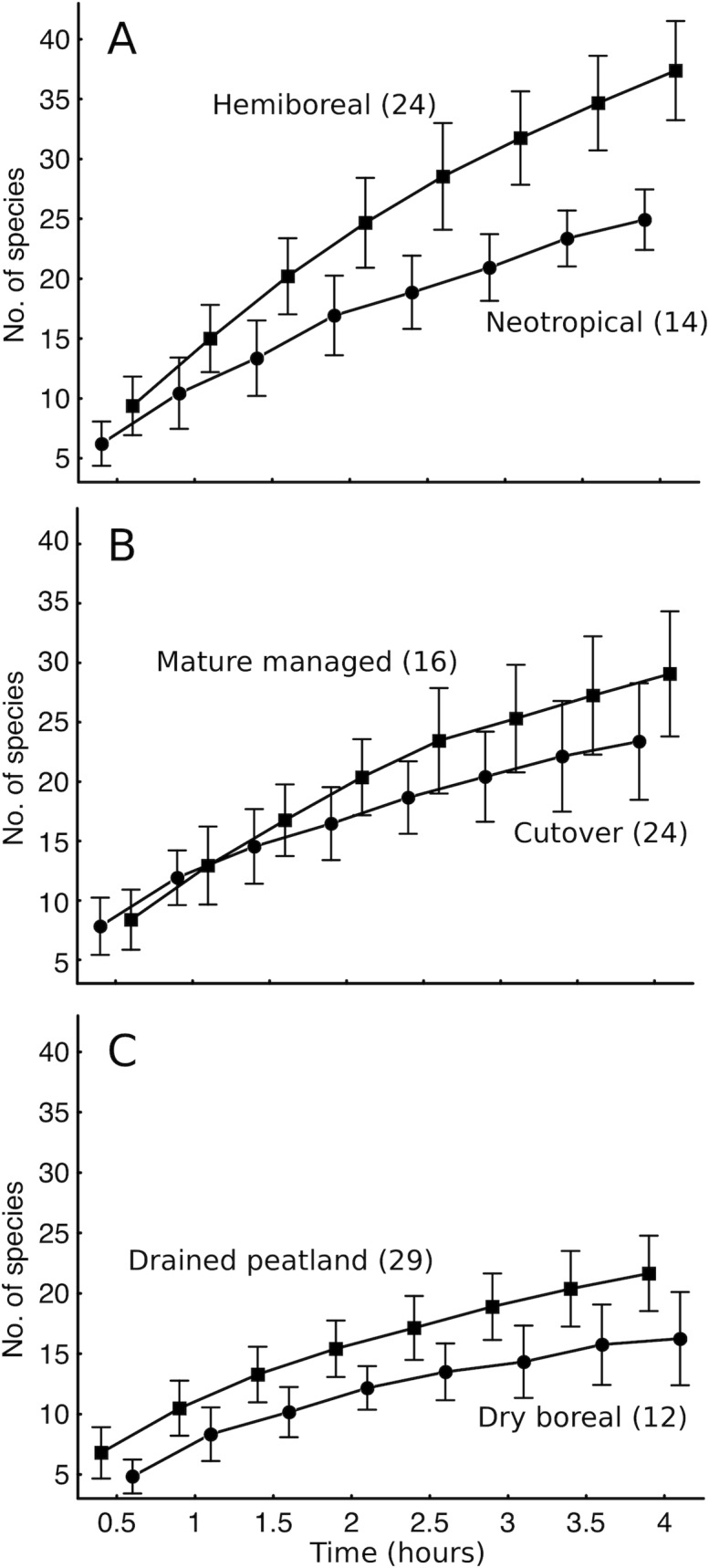
Species accumulation during 4-hour polypore surveys in 2-ha plots in different ecosystems. (A) Highly productive old growth forests in two biomes; (B) pre-harvest vs. post-harvest mixedwood stands in Estonian production forests (same site types as the hemiboreal old-growth in A); (C) Scots pine dominated mature stands on Podzols (dry boreal sites) vs. drained mixotrophic peat in Estonia. The points show arithmetic means (±SD); numbers in parentheses are sample sizes. S1 Fig illustrates the ecosystems.

**2. Target ecosystem.** The protocol can be used in open, forested, and anthropogenic land where an observer can access all relevant microhabitats or at least a stable set (e.g., 0–2 m from the forest floor). Poorly accessible microhabitats, such as tree canopies, can be added to the basic survey based on more specific protocols (e.g., [60]); supporting surveys may be also appropriate in ecosystems that have contrasting seasonal aspects (e.g. spring ephemerals or species associated with vernal pools). In our experience, the method is robust to ease of walking as long as the observer can visually scan the surroundings and optimize routes to “promising” locations. After 1–2 hours of searching effort, the species accumulation curves start levelling off in very different ecosystems (Figs 1 and 2), which does not suggest severe detectability-related bias for qualitative assemblage comparisons among ecosystems. Doubtful situations can be checked by explicitly adding extra time to increase survey completeness (see below).

In addition to extremely difficult terrains (rocky areas; thickets on >20⁰ slopes; inaccessible wetlands etc.) and restricted areas (such as crop fields), erratically fluctuating ecosystems may pose problems for using the protocol. Many species can become poorly detectable after severe disturbance, such as plants on recently mown or heavily grazed sites or emerging vegetation after wildfires. We are not aware of explicit tests on such bias, but it apparently depends on observer’s skills and should be assessed in relation to the survey goal. We also acknowledge that, due to plot area specifications (p. 4 below), the protocol is not suitable for surveying the habitat types that only occur as small patches or narrow strips.

**Fig 3:**
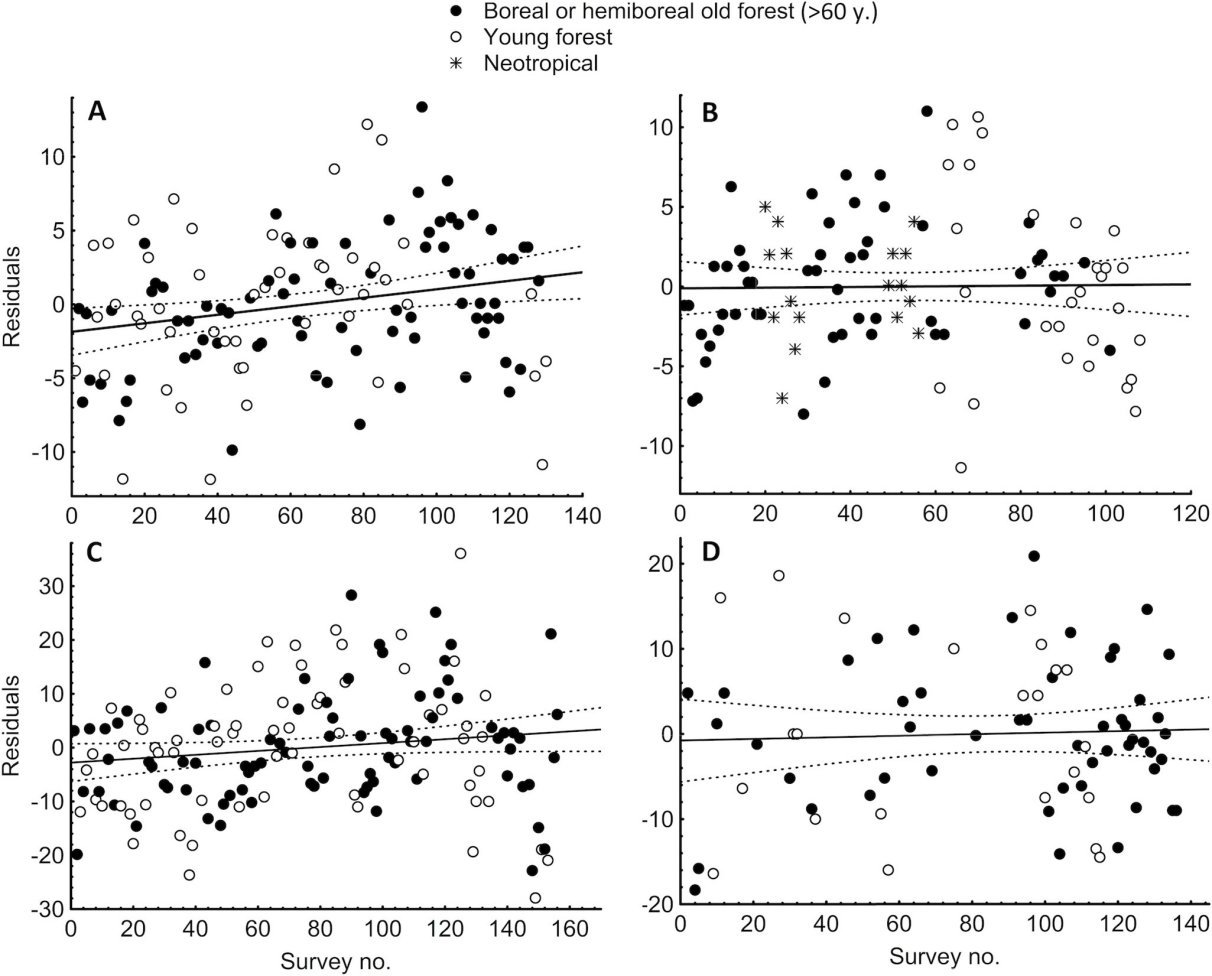
Track records of three observers using the survey protocol for four taxon groups based on >110 surveys each. (A–B) Polypores (two different observers); (C–D) lichens and bryophytes (the same observer). Linear regression (±95 CI) between the survey order and recorded species richness (y-axis, standardized by forest type) indicates a ‘learning effect’ and is statistically significant only in (A): slope 0.029 ± 0.011, *P* < 0.01.

**3. Observer qualification.** Following our protocol in the field requires three basic skills: (i) recognizing species based both on taxonomic knowledge (essential even for morphospecies based field assessments; [61]) and experience with within-species variation; (ii) recognizing different microhabitats in the studied ecosystem for exploration; and (iii) abilities for intensive focused work. With these conditions met, gaining of personal experience with the method (learning) appears to have relatively little influence on species’ discovery, compared with the variation of interest (Fig 3). Consequently, training of new experts is relatively easy: we start with a few joint surveys with an established expert to demonstrate the working routine, followed by 1–2 parallel surveys in the same plots and analyzing the differences in results together. Formal tests of among-person differences are challenging because observer tracks usually stay observable in the field and some specimens are removed by collecting. However, our preliminary assessments indicate that the numbers of species recorded and, specifically, detecting of rare and remarkable taxa are stable enough for most assemblage analyses [43]. If in doubt, observer identity should be included as a factor in data processing.

The protocol favours species identification in the field, which increases the efficiency of time use and reduces removal of rare and threatened species from the environment. Nevertheless, our experience is that full identification of even limited collected material will take at least twice the amount of field hours. Larger projects should thus incorporate additional taxonomists for lab identification, including DNA barcoding where necessary. Dividing tasks between the best field experts and those qualified in the lab provides opportunities for forming efficient and long-term survey teams.

**4. Plot area and microhabitat list.** We had four main criteria for plot size: it is fixed; large enough to represent and be monitored as an ecological community; enables practical ranking of sites for conservation; feasible for field exploration by experts during a single visit. We optimized the ecological criterion based on forests, considering reports that ca. 1 ha or more is needed for monitoring composition [62,63], including typical, but infrequent microhabitats [64], forest interior microclimate [65], and representing local species pools [45,66,67]. We then considered practical issues: parallel surveys of both species-dense and species-poor assemblages (such as lichens and birds in a common area) and, at least for the former, possibilities to directly assign sites for protection based on viable populations of multiple threatened species [44]. The 2-ha scale extracted by us has been previously recommended as a minimum for a conservation and taxonomist perspective [9]; such area frequently appears in comparative vegetation diversity studies in forests [18,68–70].

Equipped with the basic 2-ha size, we added down- and up-scalability by including microhabitat and context descriptions, respectively (p. 11–12 in the protocol). Those components are worth careful elaboration. An ideal microhabitat list could link the surveys with substrate-scale biodiversity research and environmental monitoring programs; for example, to assess the impacts of ecosystem structural changes on species and assemblages [36,39]. Up-scalability can be enhanced by spatial arrangement of plots. For example, in Estonian drained pine wetlands, we have combined plot clusters and their spacing out to multi-scale estimates of polypore species pools (as detected by fruit bodies): individual fallen trunks (on average, 2–4 species detected); 2-ha (21–22 species) and 4-ha areas (31–36 species); and landscapes (87 species) [36,43]. If such estimates are corrected for incomplete detection (see below), they can reveal species-area relationships and allow spatial modelling of species and assemblages.

**5. Survey effort.** Recording all species of a species-rich assemblage in a 2-ha area is extremely laborious and a reasonable alternative is to standardize effort at an optimal level in terms of finding rare species (when non-detection becomes random). For standardization, we have used survey time to provide experts much freedom to use their searching skills in the field. Thus, in 2-ha plots in mixed old forests >500 field hours was required for a complete list of lichens, but half of the list, including a good overview of conservation values, was obtained using our 4-hour protocol [44]. Specifically, our 4-hour standard effort considers: (i) that it usually takes 2–3 hours to detect broad, informative differences in assemblages (e.g., Figs 1 and 2) and (ii) at least 3 hours for poorly detectable species subsets to become comparable with well detectable subsets [43], and (iii) the feasibility of making two surveys per day. Similar combination of 4-hour effort per 2-ha plot has been also used by, e.g., [18,68,70]. In polypore and plant surveys, the observers A.L. and K.R. [40,41,43] have typically walked 4–7 km during a 4-hour search in a 2-ha plot (based on GPS-tracks).

**Fig 4:**
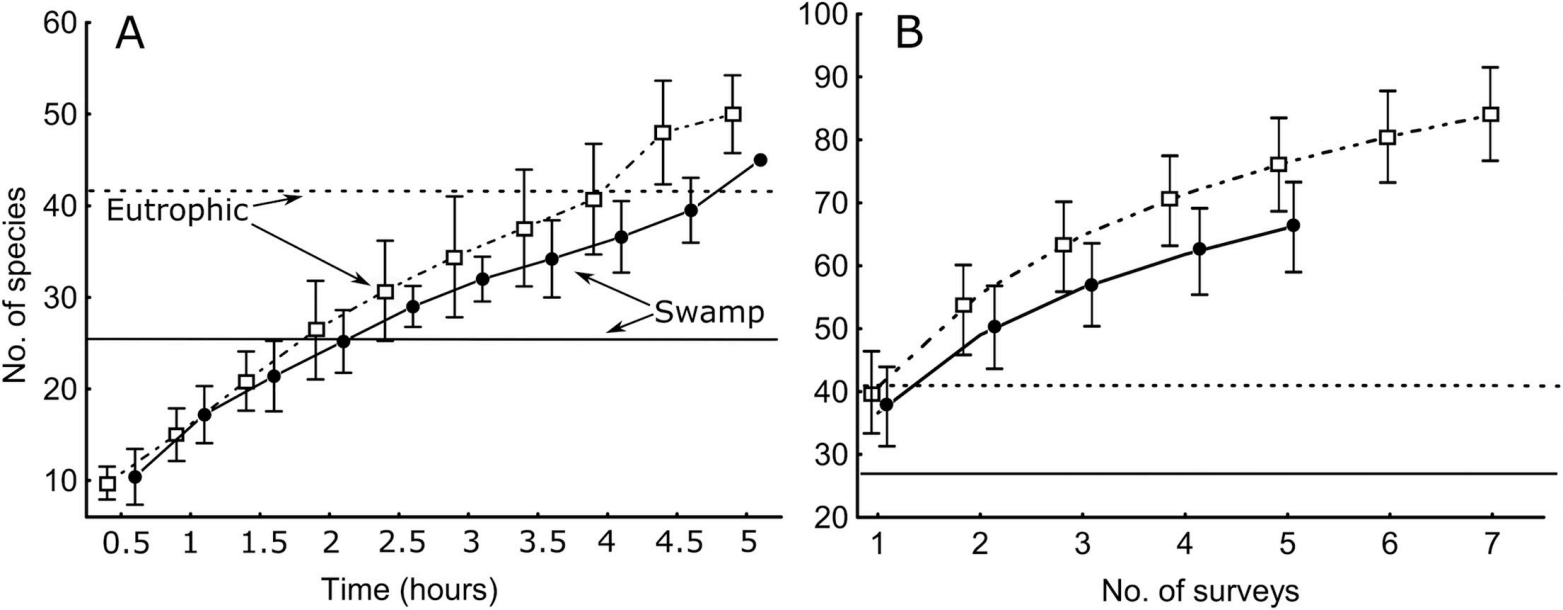
**Polypore species accumulation in 2-ha plots in two repeatedly surveyed old-growth forests in East Estonia: along with survey time (A) and in subsequent surveys, 2005–2016 (B).** The horizontal lines denote numbers of species found in the plots during many casual mycological surveys between the 1930s and 2004 [53]. In (A), the symbols denote arithmetic means (±S.D.); in the rarefaction analysis (B), the symbols are expected numbers, S(est) ±95% CI, in the pooled sample.

We have addressed two potential caveats of the fixed survey effort as follows. First, since relatively smaller fractions of all species are discovered in extremely species-rich sites (see Chapter Survey effectiveness), one can explicitly add survey time in such sites (e.g., Fig 4A). Second, in sites that appear as biodiversity hotspots according to the standard survey, we have sometimes performed separate searches of rare and threatened species or microhabitats [44,71,72]. In both cases, we retain assemblage comparisons based on standard effort, but not at the expense of missing important part of the species pool or specific species of interest. Although discouraged, the surveys can also be shorter in extremely species-poor sites if considerable time has passed without finding new species. In any case, explicit reporting of species accumulation curves is recommended.

## Survey effectiveness

1. The effectiveness of a 4-hour survey to capture *species pools at the plot scale* (2 ha) has been assessed for three taxon groups.

Repeated **polypore** surveys in two protected old-growth plots revealed that a single survey following our protocol recorded as many species as had been observed in the past 74 years during traditional mycological excursions (casual sampling; Fig 4A). In turn, each protocol-based survey detected ca. 50% of the cumulative species list of repeated surveys over 11 years in the most species-rich eutrophic forest and probably more in the less species-rich swamp forest (Fig 4B). In the latter analysis, assemblage turnover was not accounted for, so the actual effectiveness in a given year is probably higher, as also suggested by a comparative study on hyphal occurrence vs. fruit-body formation [45].

Completeness of **lichen** species lists was >70% in a microlichen group of medium detectability [39] and the lowest values found are ca. 50% in the most species-rich forests [38]. Detection rates of individuals range from 5% to 38% in studied microlichens, and probably seldom exceed 50% in even the most conspicuous species [39,66]. In **vascular plants**, >90% of field-layer species in 2-ha plot are detected in 4 hours [40,42]. For frequent species we have measured ground cover ranges that correspond to the abundance scores given during the rapid survey; this has enabled, for example, to express timber harvesting impacts also in cover units [42].

**Fig 5:**
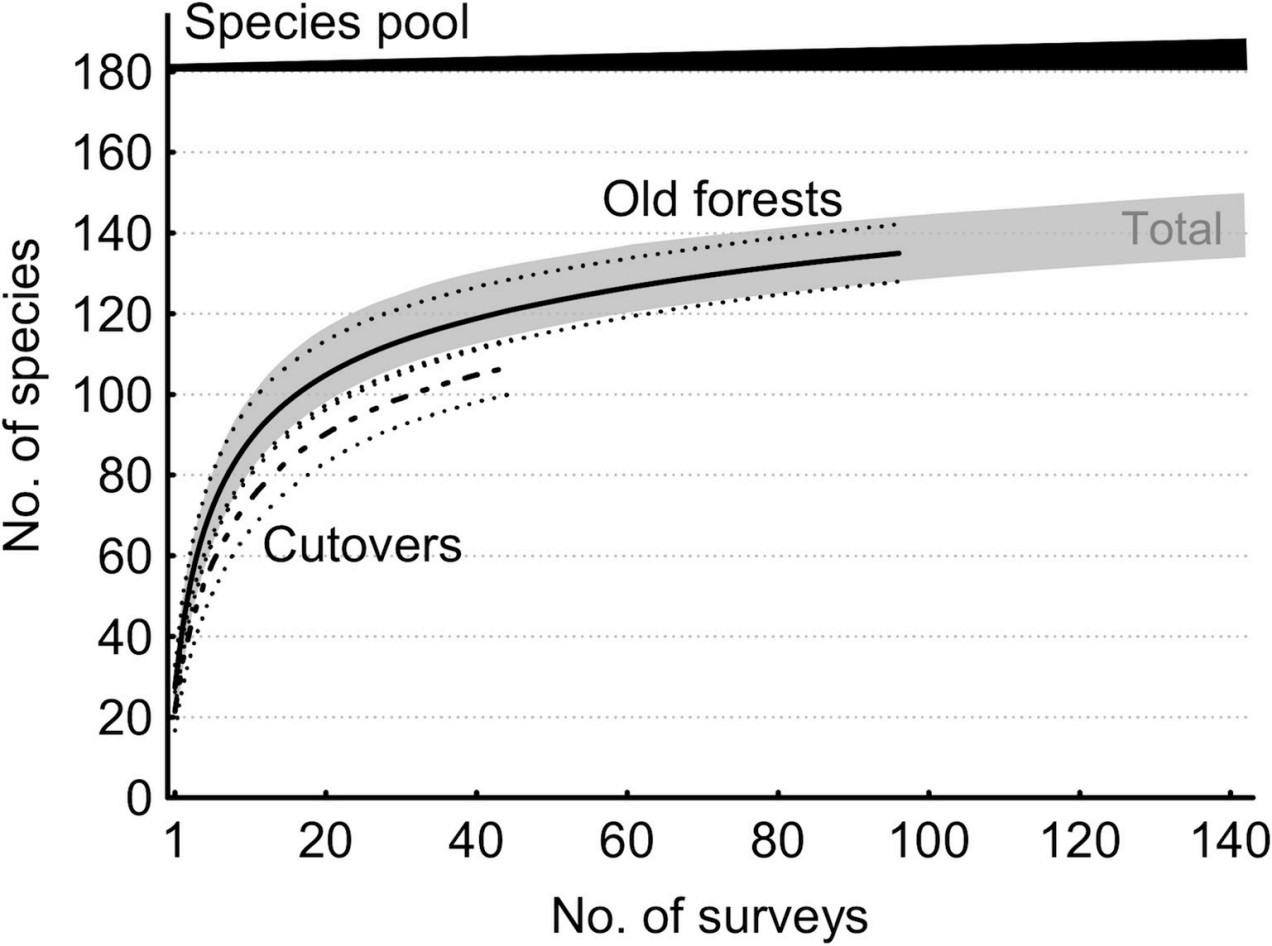
Survey-based rarefaction curves for polypore species richness in old (N= 97) and clear-cut (N=46) Estonian forests compared with the known national species pool in the surveyed forest types (black area; increasing due to 13 species added by these surveys). The curve lines denote expected numbers of species in pooled samples (S(est)) ± 95% CI; the grey area depicts a similar rarefaction for forests and clear-cuts pooled.

2. In Estonian polypores and forest lichens, we have estimated effectiveness of a protocol-based survey *program to reveal regional species pool*. The data are from setups spread out geographically and among forest types (maps in [36,39,45]), but not specifically designed to represent the country. Yet, in **polypores**, 143 surveys (i.e., 572 fieldwork hours in 286 ha) detected 78% of the 181 species previously known in the studied forest types in Estonia and improved the national list with 13 new species. The rarefaction curve was steeper in old forests than in clear-cuts and a pooled curve added little to the former (Fig 5). This indicates that all polypore species can inhabit old growth, while clear-cuts host impoverished forest assemblages – both conclusions separately reached also for calicioid fungi [39]. The old-forest curve also reveals that ca. 20 surveys in such plots can efficiently capture over half of the national species pool of polypores (Fig 5).

In **lichens**, 133 protocol-based surveys detected 70% of the 473 species that had been confirmed from forest studies prior to our program ([73]; the list revised for taxonomy). Additionally, the surveys increased the historical list by 5% – confirming 17 new species in forests (four of which were listed potentially forest-inhabiting by [73]).

**Fig 6:**
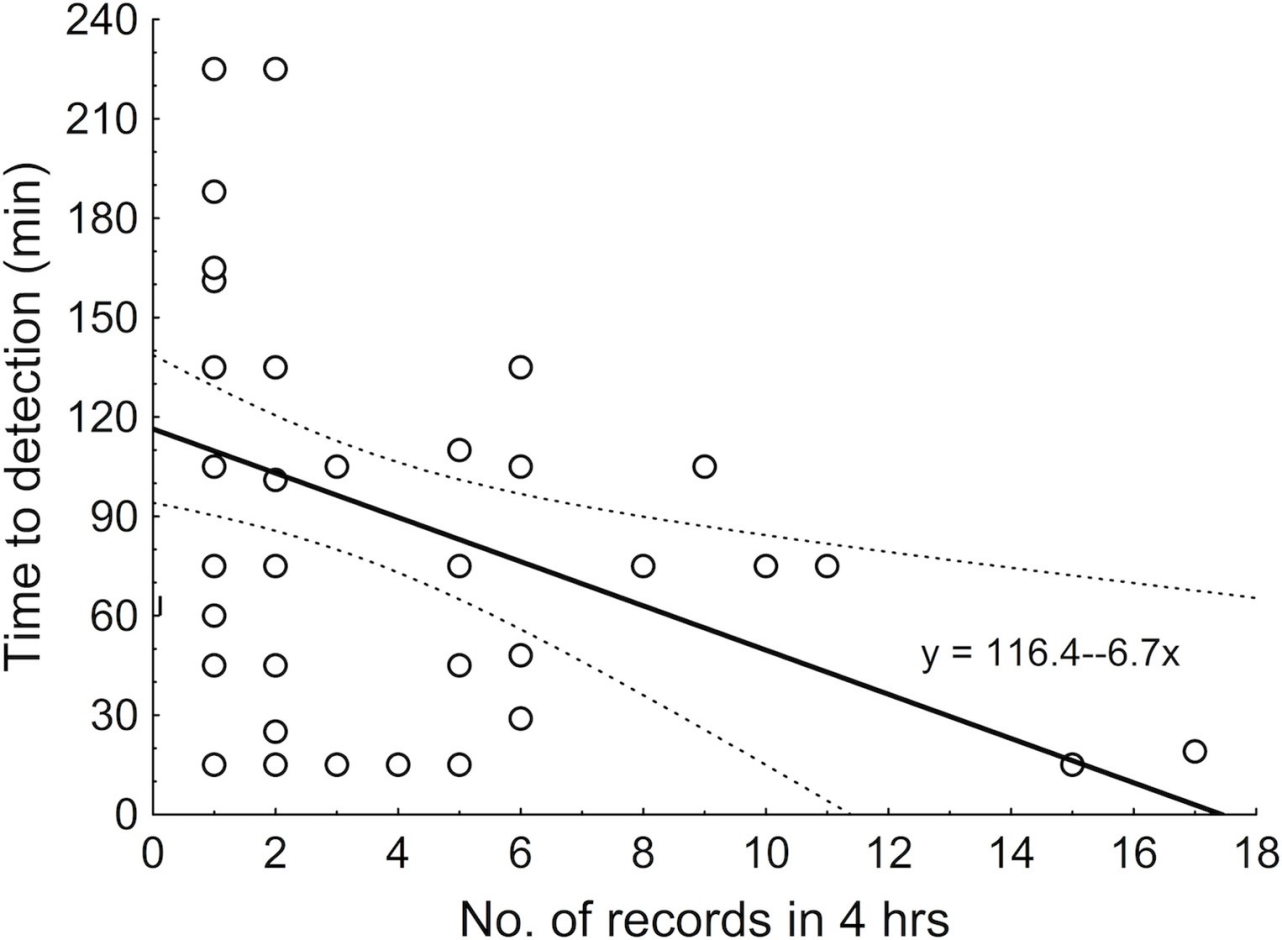
Time to detection of the polypore *Rigidoporus crocatus* in relation to its total number of records. The data are from 53 2-ha plots in Estonia where the species was detected during the 4-h surveys; no. of records refers to fallen trunks with fruit bodies found during the 4 h. The line depicts linear regression (± 95% CI; *P* < 0.001).

**Fig 7:**
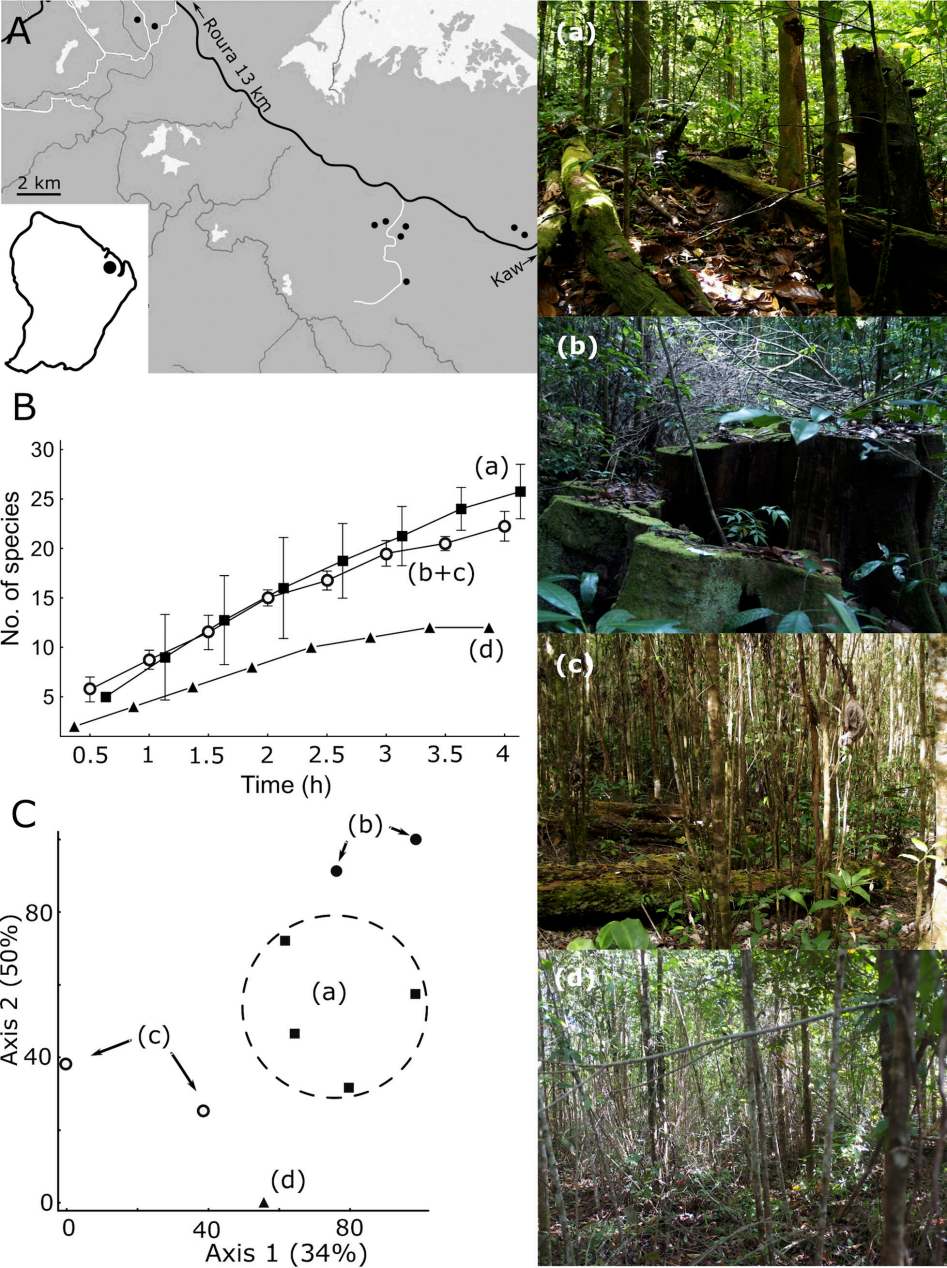
Polypore surveys in nine Neotropical *terra firma* forest sites in French Guiana over a gradient of human disturbance. (A) Location of the nine sample plots; (B) their mean species accumulation curves; (C) non-metric multidimensional scaling ordination of the assemblages (% variance explained on axis). (Right panel) The ecosystems studied: *a*, old growth (4 plots) and, *b*, selectively logged forests (2 plots); naturally regenerated slash-and-burn agriculture sites with (*c*) partial retention of forest legacies (2 plots) and, *d*, after soil scarification (1 plot). Note that (B) suggests impoverishment of treatment (d) only, but assemblage composition reveals opposite shifts after logging vs. slash-and-burn.

3. The survey effectiveness in *detecting particular species* has been measured in *Chaenotheca furfuracea* (a microlichen), for which a thorough later investigation revealed that 87% of inhabited plots were detected in 4-hour surveys [39]. A major reason for false negative records is the rarity of the species: a threatened polypore, *Rigidoporus crocatus*, was detected within one hour if >5 specimens were present, and within two hours if >1 specimens were present (Fig 6). Rare species have, however, posed even larger detection problems for traditional (non-intensive) survey approaches by taxonomists [38]. Wide variation of ‘time to detection’ at low abundances (Fig 6) indicates that this metric cannot be used as reliable proxy for local abundance.

4. Feasibility for *rapid biodiversity surveys in remote regions* is important for engaging leading taxonomists effectively to documenting unique and threatened biodiversity on a global scale. Based on our experience from many short expeditions to abroad, our protocol can provide standard documentation of regional biodiversity and its variation during such expeditions. As an example, polypore sampling by K.R. in French Guiana in November 2013: (i) captured the major pattern of human disturbance in tropical forests (see [74]) – that species richness may remain relatively stable but assemblage composition changes profoundly (Fig 7); (ii) provided the first regional species list comprising 87 species (S3 Table), well exceeding the 73 species listed in the neighbouring Guyana [75].

## Opportunities and applications of the data

Our published studies that are at least partly based on the protocol (18 in total; most listed in Table 1) illustrate four main opportunities of using the data.

1. **Broad comparative assessments of species pools**, based on their cost-efficient documenting at scales relevant for management in various terrestrial ecosystems. Such properties enable *snapshot assessments of biodiversity responses to disturbances* across multiple taxon groups [41], or along broad environmental [43] and geographical gradients [47]. The species abundance data collected enable estimating most assemblage metrics that are not based on exact numbers of individuals; however, several jackknife and other species richness estimators that only use the abundances of rare species [76] are also possible to calculate. We have estimated, for example, species richness from sample-based rarefaction (Fig 5), and species turnover and indicator value (e.g., [41]), and we recognize the possibilities of the species co-occurrence matrices for estimating ‘dark diversity’ and its derivates of management value [77]. The species data can also be organized by microhabitats, with an application to assess or predict impacts of microhabitat loss on biodiversity (e.g., [39,48]). When the assemblage data are supplemented with habitat measurements (Protocol: p. 12), *key factors of the assemblages and their variation* can be analysed. For example, an analysis of artificially drained forests based on >1000 species [41] served as a key for distinguishing those forests as novel ecosystems with specific options for sustainable management and restoration [78].

2. **Assessment of infrequent, poorly known or special species** that are recorded due to allocating field effort to full species lists and all microhabitats. Those properties can be enhanced by sampling poorly studied ecosystems (e.g., [38]) and adding field effort in certain places considering the discovery time of species of interest (see above). By applying the area-based abundance estimates (including absences) to habitat areas, the method has become the main information source for estimating *regional conservation status of species* in the taxon groups surveyed (e.g., [39,79]). Habitat measurements enable *modelling of (rare) species’ ecological requirements* [36] and, potentially, prediction in space and time. We have repeatedly found *undescribed taxa* for further taxonomic work, which benefits from the ecological context described [33,80,81]. The data also allow assessment of some biodiversity-based goods, such as wild fruits [42].

3. **Libraries of sites for ecosystem assessment and monitoring**, both at the scale of sites, habitat types and regional species pools (e.g., Figs 4 and 5). We have kept the field procedure simple, independent of specific equipment and usable across projects, which thus represent modules of a general libary. Such library considerably expands the opportunities for (i) establishing *reference values of biodiversity* to be used for assessing individual sites, including their contribution to existing reserve networks [44,82,83]; (ii) comparing biodiversity impacts of management systems studied in different projects [43,46,47]; (iii) assessing *temporal changes in repeatedly sampled locations* [42]. The data can be integrated into spatial models of ecosystem quality or composite indices, for example, the Index of Favourable Conservation Status [84].

4. **Site mapping for in-depth studies on populations and assemblages**. We have used the protocol for a first step (*coarse-scale distribution mapping*) for population studies on rare and poorly detectable species [72], and combined it with molecular sampling of fungal assemblages [45]. Such intensive follow-up studies, in turn, have provided estimates of the effectiveness of the protocol. The standardized field effort meets a crucial condition for studying species co-occurrence patterns, including distinguishing of indicators for monitoring and management [43].

## Discussion

We have extensively documented the performance of a simple survey protocol for full assemblages of species-rich inconspicuous land organisms to address taxonomic, ecological and conservation topics. Our effort addresses previous calls to increase the efficiency of biodiversity research: to establish links between the disciplines that use taxonomic expertise [26], provide explicit biodiversity information for management [85], include all taxa, with specific attention on rarities [86], and to have common indicators across ecosystem types [3]. Technically, we have focused on a few simple rules (fixed area; fixed effort; task prioritization), while allowing freedom in terms of sampling designs (comparative studies; sampling along time; experimental) to increase the range of study questions that can be answered [87]. This is a difference from several previous attempts to standardize biodiversity monitoring that place much attention on establishing standard sampling designs (S4 Table). Highly standardized frameworks have been criticised for providing sub-optimal and costly solutions for problem-driven ecological monitoring [88]. Thus, while addressing the general trend of diversification in biodiversity monitoring [89], our approach retains the possibilities to combine datasets from different projects into a standard biodiversity database and perform formal analyses using large samples. For example, in Estonia, our database has been instrumental in transforming qualitative conservation assessments of species (based on diverse ‘evidence’) into much more explicit calculations [38,72].

Cost-effectiveness of any ecological survey protocol can be also criticized based on the entities it measures, given that data collection should follow from specific questions [88]. While agreeing with this principle, we argue for universal value of full biodiversity descriptions, particularly in our era of global biodiversity loss and transformation. For that basic reason we have looked for cost-effective survey methods to record full species assemblages, especially of large, so-far poorly studied taxon groups; a part of the cost-effectiveness being the possibility to survey multiple taxon groups in the same areas. Such explicit focus on full assemblages in common areas, while keeping survey costs reasonable, is another difference from most standard biodiversity monitoring schemes proposed (S4 Table). Thus, we have avoided subjectively selecting subsets of species or microhabitats (e.g., [10,11,90,91]) and we have allocated the effort from exhaustive sampling and abundance measurements (e.g., [92]) toward fixed-time searching for rare, sparsely distributed and poorly known taxa.

A key to our approach was the question how to best use the expertise of field taxonomists, with implications to their training. It is known that taxonomists who participate in ecological sampling of inconspicuous taxa can effectively find undiscovered species [93,94] and identification skills are crucial to reduce false positive records [95,96]. A feature that we have explicitly added is that, because the plot size is fixed at an ecosystem scale relevant for conservation, additional skills are needed for effective searching of new species within the plot. Field tests of how those skills vary among experts are technically challenging and yet to be completed; however, we have shown that, within person, long-term acquisition of experience affects results relatively little. A lichen study found that documenting assemblage differences in time-constrained surveys are repeatable even for technicians [97]. We thus hypothesize that, given enough time, differences in species lists attributable to the working routines of trained experts will converge to levels acceptable for many study questions. These differences are further reduced using clear rules for collecting specimens difficult to identify in the field, and the lab work with those specimens designed as a separate activity in actual projects.

Perhaps the most challenging issue with any biodiversity survey is the repeatability of detecting rare species [98,99]. Indeed, the discovery time varies much at low abundances (Fig 6), although one can argue that, for conservation purposes, detecting viable local populations is the priority task. We have enhanced the discovery of rare species by using established experts (cf., [100,101]) and, more arguably, by increasing plot size (cf., [102]). Supporting the latter, we have documented that our 2-ha plots are effective for detecting (by fruit bodies) the presence of filamentous fungal species, which at small scale mostly remain hidden within the substrate and visually unrecognizable [45]. Acknowledging that specimens are more frequently missed in large plots [99], we have prioritized species list above abundance estimates. In terms of species distribution modelling, it refers to focusing on reliable larger-scale presences instead of smaller-scale absences, because the former are more limiting in the case of rare species [103]. Due to the missed specimens, we have also re-scaled abundance estimates to increasingly broad categories, which has an additional advantage of normalizing the naturally hyperbolic distributions of species in assemblages [104].

There are obvious limits to standardizing of biodiversity survey methods, even in the specific case of visually recognizable, but inconspicious, land organisms addressed by us. Perhaps most importantly, our protocol is not applicable in the areas where the field expert cannot move around freely. Comparable methods are nevertheless much needed for conservation, particularly under the severe funding constraints in developing countries [105]. Our experience demonstrates that widely usable standard protocols can be developed, particularly as parts of structured survey programs, where the limitations of a particular protocol can be resolved through other activities of the program. By necessity, we have for example subsampled the fixed 2-ha plots for soil organisms or accurate vegetation cover estimates, or embedded the plots within larger bird survey areas [42,106]. A long-term problem is that taxonomy and identification keys of megadiverse taxon groups are likely to change, undermining repeatability of species lists [107]. To address this, the data should remain traceable by individual taxa [108] and thoughtfully supported with collections.

Variation in the detectability of species and specimens stands out as the major issue for further research regarding our protocol and other biodiversity survey methods that allow considerable freedom of field experts. For example, observer bias might be estimated and partly corrected by using paired or multiple experts in the field [96,109]. Such an approach might be more effective than doubling survey time by the same person if individual experts are biased toward missing certain species. To reduce the possibly smaller efficiency of early-career experts (e.g., [110]), standard training programs might be elaborated. Detectability assessments should be carried out comparatively in different ecosystems and, specifically, in those cases where they may confound with the impacts under study. The actual impacts of difficult terrain on survey results should be estimated and possibilities to take that into account (e.g., by extended surveying) should be considered. We also look forward in using our protocol in complex landscapes, such as in agricultural mosaics and the countryside where we have carried out only preliminary surveys.

## Supporting information

S1 Fig Examples of contrasting forest systems surveyed using the fixed-area-fixed-effort approach and analysed on Fig 1. (PDF)

S1 Table The study sites, their key characteristics and references to the published papers using the polypore data from these sites. (XLSX)

S2 Table Data tables of the analyses. (XLSX)

S3 Table List of taxa collected during nine polypore inventories in French Guiana; and numbers of their voucher specimens in TU fungarium. (XLSX)

S4 Table Survey methods used in plant and fungal monitoring schemes. (PDF)

## References

[1] Visconti P, Bakkenes M, Baisero D, Brooks T, Butchart SH, Joppa L, et al Projecting global biodiversity indicators under future development scenarios. Conserv Lett. 2016; 9: 5–13.

[2] Mora C, Tittensor DP, Adl S, Simpson AG, Worm B. How many species are there on Earth and in the ocean? PLoS Biol. 2011; 9: e1001127 10.1371/journal.pbio.1001127 21886479 10.1371/journal.pbio.1001127 PMC3160336

[3] Feld CK, Martins da Silva P, Paulo Sousa J, De Bello F, Bugter R, Grandin U, et al Indicators of biodiversity and ecosystem services: a synthesis across ecosystems and spatial scales. Oikos. 2009; 118: 1862–1871.

[4] Maes J, Liquete, Teller A, Erhard M, Paracchini ML, Barredo JI, et al An indicator framework for assessing ecosystem services in support of the EU Biodiversity Strategy to 2020. Ecosyst Serv. 2016; 17: 14–23.

[5] Römbke JC, Gardi C, Creamer R, Miko L. Soil biodiversity data: Actual and potential use in European and national legislation. Appl Soil Ecol. 2016; 97: 125–133.

[6] Cardoso P, Erwin TL, Borges PA, New TR. The seven impediments in invertebrate conservation and how to overcome them. Biol Conserv. 2011; 144:2647–2655.

[7] San-Miguel-Ayanz J, Parviainen J, Schuck A, Bozzano M, Estreguil C, Koskela J, et al Criterion 4: maintenance, conservation and appropriate enhancement of biological diversity in forest ecosystems State of Europe’s Forests 2011: status and trends in sustainable forest management in Europe Forest Europe, UN Economic Commission for Europe and FAO Oslo, 2011; pp 65–97.

[8] Kull T, Sammul M, Kull K, Lanno K, Tali K, Gruber B, et al Necessity and reality of monitoring threatened European vascular plants. Biodivers Conserv. 2008; 17: 3383–3402.

[9] Hunter ML, Webb SL. Enlisting taxonomists to survey poorly known taxa for biodiversity conservation: a lichen case study. Conserv Biol. 2002; 16: 660–665.

[10] Mueller GM, Schmit JP, Hubndorf SM, Ryvarden L, O'Dell TE, Lodge DJ, Leacock PR, Mata M, Umania L, Czederpiltz DL. Recommended protocols for sampling macrofungi In: Mueller GM, editor. Biodiversity of fungi: inventory and monitoring methods. Elsevier Academic Press, Boston; 2004 pp. 168–172.

[11] Ah-Peng C, Wilding N, Kluge J, Descamps-Julien B, Bardat J, Chuah-Petiot M, Strasberg D, Hedderson TA. Bryophyte diversity and range size distribution along two altitudinal gradients: continent vs. island. Acta Oecol. 2012; 42: 58–65.

[12] Silvertown J. A new dawn for citizen science. Trends Ecol Evol. 2009; 24: 467–471. 10.1016/j.tree.2009.03.017 19586682 10.1016/j.tree.2009.03.017

[13] Costello MJ, May RM, Stork NE. Can we name Earth's species before they go extinct? Science. 2013; 339: 413–416. 10.1126/science.1230318 23349283 10.1126/science.1230318

[14] Mueller GM, Bills GF, Foster MS, editors. Biodiversity of fungi: inventory and monitoring methods. Boston, MA: Elsevier Academic Press; 2004.

[15] Hortal J, de Bel F, Diniz-Fil JAF, Lewinsoh TM, Lob JM, Ladle RJ. Seven shortfalls that beset large-scale knowledge of biodiversity. Annu Rev Ecol Evol Syst. 2015; 46: 523–549.

[16] Sutherland WJ, editor. Ecological census techniques: a handbook. Cambridge: Cambridge University Press; 2006.

[17] Edwards TC Jr, Cutler DR, Geiser L, Alegria J, McKenzie D. Assessing rarity of species with low detectability: lichens in Pacific Northwest forests. Ecol Appl. 2004; 14: 414–424.

[18] Newmaster SG, Belland RJ, Arsenault A, Vitt DH, Stephens TR. The ones we left behind: comparing plot sampling and floristic habitat sampling for estimating bryophyte diversity. Divers Distrib. 2005: 11; 57–72.

[19] Bertuol‐Garcia D, Morsello C, N. El‐Hani C, Pardini R. A conceptual framework for understanding the perspectives on the causes of the science–practice gap in ecology and conservation. Biol Rev. 2018; 93: 1032–1055. 10.1111/brv.12385 29160024 10.1111/brv.12385

[20] Thompson W, editor. Sampling rare or elusive species: concepts, designs, and techniques for estimating population parameters. Washington, DC: Island Press; 2013.

[21] Bohmann K, Evans A, Gilbert MTP, Carvalho GR, Creer S, Knapp M. Environmental DNA for wildlife biology and biodiversity monitoring. Trends Ecol. Evol. 2014; 29: 358–367. 10.1016/j.tree.2014.04.003 24821515 10.1016/j.tree.2014.04.003

[22] Thomsen PF, Willerslev E. Environmental DNA – An emerging tool in conservation for monitoring past and present biodiversity. Biol Conserv. 2015; 183: 4–8.

[23] Stem C, Margoluis R, Salafsky N, Brown M. Monitoring and evaluation in conservation: a review of trends and approaches. Conserv Biol. 2005; 19: 295–309.

[24] Ji Y, Ashton L, Pedley SM, Edwards DP, Tang Y, Nakamura A, et al Reliable, verifiable and efficient monitoring of biodiversity via metabarcoding. Ecol Lett. 2013; 16: 1245–1257. 10.1111/ele.12162 23910579 10.1111/ele.12162

[25] Geiger MF, Moriniere J, Hausmann A, Haszprunar G, Wägele W, Hebert PD, et al Testing the Global Malaise Trap Program – How well does the current barcode reference library identify flying insects in Germany? Biodivers Data J. 2016; 4: e10671. 10.3897/BDJ.4.e10671 PMC5136679 27932930

[26] Halme P, Kuusela S, Juslén A. Why taxonomists and ecologists are not, but should be, carpooling? Biodivers Conserv. 2015; 24: 1831–1836.

[27] Pirolli P. Information foraging theory: Adaptive interaction with information New York: Oxford University Press; 2007.

[28] Ehinger KA, Wolfe JM. When is it time to move to the next map? Optimal foraging in guided visual search. Atten Percept Psychophys. 2016; 78: 2135–2151. 10.3758/s13414-016-1128-1 27192994 10.3758/s13414-016-1128-1 PMC5014635

[29] IUCN Standards and Petitions Subcommittee. Guidelines for Using the IUCN Red List Categories and Criteria Version 13. 2017. Available from: http://wwwiucnredlistorg/documents/RedListGuidelines.pdf

[30] Guisan A, Thuiller W. Predicting species distribution: offering more than simple habitat models. Ecol Lett. 2005; 8: 993–1009. 10.1111/j.1461-0248.2005.00792.x 34517687

[31] Lonsdale D, Pautasso M, Holdenrieder O. Wood-decaying fungi in the forest: conservation needs and management options. Eur J For Res. 2008: 127; 1–22.

[32] Junninen K, Komonen A. Conservation ecology of boreal polypores: a review. Biol Conserv. 2011; 144: 11–20.

[33] Runnel K, Põldmaa K, Lõhmus A. ‘Old-forest fungi’are not always what they seem: the case of Antrodia crassa. Fungal Ecol. 2014; 9: 27–33.

[34] Hallingbäck T. Working with Swedish cryptogam conservation. Biol Conserv. 2007; 135: 334–340.

[35] Dahlberg A, Mueller GM. Applying IUCN red-listing criteria for assessing and reporting on the conservation status of fungal species. Fungal Ecol. 2011; 4: 147–162.

[36] Runnel K, Lõhmus A. Deadwood-rich managed forests provide insights into the old-forest association of polypores. Fungal Ecol. 2017; 27: 155–167.

[37] Nelson GC, Bennett E, Berhe AA, Cassman K, DeFries R, Dietz T, Dobermann A, Dobson A, Janetos A, Levy M, Marco D. Anthropogenic drivers of ecosystem change: an overview. Ecol Soc. 2006; 11: 29.

[38] Lõhmus P, Lõhmus A. The importance of representative inventories for lichen conservation assessments: the case of *Cladonia norvegica* and *C parasitica*. Lichenologist. 2009; 41: 61–67.

[39] Lõhmus A, Lõhmus P. Old-forest species: the importance of specific substrata vs stand continuity in the case of calicioid fungi. Silva Fennica. 2011; 45: 1015–1039.

[40] Lõhmus A, Kull T. Orchid abundance in hemiboreal forests: stand-scale effects of clear-cutting, green-tree retention, and artificial drainage. Can J For Res. 2011; 41: 1352–1358.

[41] Remm L, Lõhmus P, Leis M, Lõhmus A. Long-term impacts of forest ditching on non-aquatic biodiversity: conservation perspectives for a novel ecosystem. PLoS ONE. 2013; e63086 10.1371/journal.pone.0063086 23646179 10.1371/journal.pone.0063086 PMC3639956

[42] Lõhmus A, Remm L. Disentangling the effects of seminatural forestry on an ecosystem good: bilberry (*Vaccinium myrtillus*) in Estonia. For Ecol Manage. 2017; 404: 75–83.

[43] Lõhmus A, Runnel K. Assigning indicator taxa based on assemblage patterns: beware of the effort and the purpose! Biol Conserv. 2018; 219: 147–152.

[44] Lõhmus P, Leppik E, Motiejunaite J, Suija A, Lõhmus A. Old selectively cut forests can host rich lichen communities – lessons from an exhaustive field survey. Nov Hedwigia. 2012; 95: 493–515.

[45] Runnel K, Tamm H, Lõhmus A. Surveying wood-inhabiting fungi: Most molecularly detected polypore species form fruit-bodies within short distances. Fungal Ecol. 2015; 18: 93‒99.

[46] Tullus T, Rosenvald R, Leis M, Lõhmus P. Impacts of shelterwood logging on forest bryoflora: Distinct assemblages with richness comparable to mature forests. For Ecol Manage. 2018; 411: 67–74.

[47] Lõhmus P, Lõhmus A, Hämäläinen A. Rapid legacy-dependent succession of lichen assemblages after forest fires: insights from two boreal regions. J Veg Sci. 2018; 29: 200–212.

[48] Hämäläinen A, Kouki J, Lõhmus P. Potential biodiversity impacts of forest biofuel harvest: lichen assemblages on stumps and slash of Scots pine. Can J For Res. 2015; 45: 1239–1247.

[49] Lõhmus A, Kraut A. Stand structure of hemiboreal old-growth forests: Characteristic features, variation among site types, and a comparison with FSC-certified mature stands in Estonia. For Ecol Manage. 2010; 260: 155–165.

[50] Parrotta JA, Knowles OH, Wunderle JM Jr. Development of floristic diversity in 10-year-old restoration forests on a bauxite mined site in Amazonia. For Ecol Manage. 1997; 99: 21–42.

[51] Nascimento HE, Laurance WF. Total aboveground biomass in central Amazonian rainforests: a landscape-scale study. For Ecol Manage. 2002; 168: 311–321.

[52] Colwell RK. EstimateS: Statistical estimation of species richness and shared species from samples. Version 9 User's Guide and application. 2013; Available at: http://purloclcorg/estimates

[53] Parmasto E. Distribution Maps of Estonian Fungi, III Pore Fungi. Tartu: Institute of Zoology and Botany of the Estonian Agricultural University; 2004.

[54] Parmasto E, Kalamees K, Kalmeti U, Parmasto I, Raitviir A, Vaasma M. Fungi of the Järvselja Primeval Forest Reserve In: Kasesalu K, editor. Järvselja Primeval Forest Reserve. Tartu: Eesti Metsaselts; 2004 pp. 60–137. (In Estonian with English summary)

[55] Lõhmus A. Factors of species-specific detectability in conservation assessments of poorly studied taxa: the case of polypore fungi. Biol Conserv. 2009; 142: 2792–2796.

[56] Renvall P, Junninen K. *Rigidoporus crocatus* recollected in Finland, plus new records of other rare polypores (Basidiomycetes). Karstenia. 1999; 39: 33–35.

[57] McCune B, Mefford MJ. PC-ORD Multivariate Analysis of Ecological Data, Version 6.0 for Windows. 2011.

[58] Lõhmus A. Relative abundance of amphibians and reptiles in forests and clear-cuts of different types. Year-book of the Estonian Naturalists’ Society. 2006; 84: 207–217.

[59] Kadlec T, Tropek R, Konvicka M. Timed surveys and transect walks as comparable methods for monitoring butterflies in small plots. J Insect Conserv. 2012; 16: 275–280.

[60] Gradstein SR, Nadkarni NM, Krömer T, Holz I, Nöske N. A protocol for rapid and representative sampling of epiphyte diversity of tropical rain forests. Selbyana. 2003; 24: 87–93.

[61] Giordani P, Brunialti G, Benesperi R, Rizzi G, Frati L, Modenesi P. Rapid biodiversity assessment in lichen diversity surveys: implications for quality assurance. J Environ Monit. 2009; 11: 730–735. 10.1039/b818173j 19557222 10.1039/b818173j

[62] Busing RT, White PS. Effects of area on old-growth forest attributes: implications for the equilibrium landscape concept. Landsc Ecol. 1993; 8: 119–126.

[63] Ranius T, Jonsson BG, Kruys N. Modeling dead wood in Fennoscandian old-growth forests dominated by Norway spruce. Can J For Res. 2004; 34: 1025–1034.

[64] Zenner EK, Peck JE. Characterizing structural conditions in mature managed red pine: spatial dependency of metrics and adequacy of plot size. For Ecol Manage. 2009; 257: 311–320.

[65] Scherer‐Lorenzen M, Potvin C, Koricheva J, Schmid B, Hector A, Bornik Z, et al The design of experimental tree plantations for functional biodiversity research In: Scherer‐Lorenzen M, Körner C, Schulze ED, editors. Forest Diversity and Function: Temperate and Boreal Systems. Ecological Studies 176. Berlin: Springer 2005 pp. 347–376.

[66] Lieberman D, Lieberman M, Peralta R, Hartshorn GS. Tropical forest structure and composition on a large-scale altitudinal gradient in Costa Rica. J Ecol. 1996; 84: 137–152.

[67] Humphrey JW, Davey S, Peace AJ, Ferris R, Harding K. Lichens and bryophyte communities of planted and semi-natural forests in Britain: the influence of site type, stand structure and deadwood. Biol Conserv. 2002; 107: 165–180.

[68] Johansson P, Gustafsson L. Red-listed and indicator lichens in woodland key habitats and production forests in Sweden. Can J For Res. 2001; 31: 1617–1628.

[69] Burke DM, Elliott KA, Holmes SB, Bradley D. The effects of partial harvest on the understory vegetation of southern Ontario woodlands. For Ecol Manage. 2008; 255: 2204–2212.

[70] McMullin RT, Thompson ID, Newmaster SG. Lichen conservation in heavily managed boreal forests. Conserv Biol. 2013; 27: 1020–1030. 10.1111/cobi.12094 23869621 10.1111/cobi.12094

[71] Lõhmus P, Turja K, Lõhmus A. Lichen communities on treefall mounds depend more on root-plate than stand characteristics. For Ecol Manage. 2010; 260: 1754–1761.

[72] Lõhmus A, Suija A, Lõhmus P. Intensive local surveys can complement rapid survey techniques to provide insights into the population size and ecology of lichenised fungi. Fungal Ecol. 2013; 6: 449–452.

[73] Lõhmus P. Composition and substrata of forest lichens in Estonia: a meta-analysis. Folia Cryptog Est. 2003; 40: 19–38.

[74] Stork NE, Srivastava DS, Eggleton P, Hodda M, Lawson G, Leakey RRB, et al Consistency of effects of tropical‐forest disturbance on species composition and richness relative to use of indicator taxa. Conserv Biol. 2017; 31: 924–933. 10.1111/cobi.12883 27982481 10.1111/cobi.12883

[75] Aime MC, Henkel TW, Ryvarden L. Studies in neotropical polypores 15: new and interesting species from Guyana. Mycologia. 2003: 95; 614–619. 21148970 10.1080/15572536.2004.11833065

[76] Gotelli NJ, Colwell RK. Estimating species richness In: Magurran AE, McGill BJ, editors. Biological diversity: Frontiers in measuring biodiversity. Oxford: Oxford University Press; 2011 pp. 39 –54.

[77] Lewis RJ, Szava-Kovats R, Pärtel M. Estimating dark diversity and species pools: An empirical assessment of two methods. Methods Ecol Evol. 2015; 7: 104–113.

[78] Lõhmus A, Remm L, Rannap R. Just a ditch in forest? Reconsidering draining in the context of sustainable forest management. BioScience. 2015; 65: 1066‒1076.

[79] Lõhmus A, Vunk E, Runnel K. Conservation management for forest fungi in Estonia: the case of polypores. Folia Cryptogam Est. 2018; 55: 79–89.

[80] Runnel K, Ryvarden L. *Polyporus minutosquamosus* sp nov from tropical rainforests in French Guiana with a key to neotropical species of Polyporus (Polyporaceae, Basidiomycota). Nov Hedwigia. 2016; 103: 339‒347.

[81] Suija A, Suu A, Lõhmus P. Substrate specificity corresponds to distinct phylogenetic lineages: the case of *Chaenotheca brunneola*. Herzogia. 2016; 29: 355–363.

[82] Saar I, Lõhmus A, Parmasto E. Mycobiota of the Poruni old-growth forest (Estonia, Puhatu Nature Reserve). Forestry Studies. 2007; 47: 71–86.

[83] Suija A, Lõhmus P, Jüriado I. The lichen biota of the Agusalu and Puhatu reserves (Estonia): the first overview. Forestry Studies. 2007; 47: 99–116.

[84] Helm A, Zobel M, Moles AT, Szava‐Kovats R, Pärtel M. Characteristic and derived diversity: implementing the species pool concept to quantify conservation condition of habitats. Divers Distrib. 2015; 21: 711–721.

[85] Balmford A, Gaston KJ. Why biodiversity surveys are good value. Nature. 1999; 398: 204.

[86] McRae L, Deinet S, Freeman R. The diversity-weighted living planet index: controlling for taxonomic bias in a global biodiversity indicator. PloS ONE. 2017; 12: e0169156 10.1371/journal.pone.0169156 28045977 10.1371/journal.pone.0169156 PMC5207715

[87] De Palma A, Sanchez-Ortiz K, Martin PA, Chadwick A, Gilbert G, Bates AE, et al Challenges with inferring how land-use affects terrestrial biodiversity: Study design, time, space and synthesis. Adv Ecol Res. 2018; 58: 163–199.

[88] Lindenmayer DB, Likens GE. Effective ecological monitoring. London, Washington DC: Earthscan; 2010.

[89] Marsh DM, Trenham PC. Current trends in plant and animal population monitoring. Conserv Biol. 2008; 22: 647–655. 10.1111/j.1523-1739.2008.00927.x 18445076 10.1111/j.1523-1739.2008.00927.x

[90] Asta J, Erhardt W, Ferretti M, Fornasier F, Kirschbaum U, Nimis PL, Purvis OW, Pirintsos S, Scheidegger C, van Haluwyn C, Wirth V. Mapping lichen diversity as an indicator of environmental quality In: Nimis PL, Scheidegger C, Wolseley P, editors. Monitoring with Lichens- Monitoring Lichens. Kluwer Academic Publisher, Netherlands; 2002 pp. 273–279.

[91] Casanovas P, Lynch HJ, Fagan WF. Using citizen science to estimate lichen diversity. Biol Cons. 2014; 171: 1–8.

[92] Dengler J, Jansen F, Glöckler F, Peet RK, De Cáceres M, Chytrý M, Ewald J, Oldeland J, Lopez‐Gonzalez G, Finckh M, Mucina L. The Global Index of Vegetation‐Plot Databases (GIVD): a new resource for vegetation science. Journal of Vegetation Science. 2011; 22: 582–597.

[93] Abrego N, Salcedo I. Taxonomic gap in wood-inhabiting fungi: identifying understudied groups by a systematic survey. Fungal Ecol. 2015; 15: 82–85.

[94] Ellis CJ, Coppins BJ. Taxonomic survey compared to ecological sampling: are the results consistent for woodland epiphytes? Lichenologist. 2017; 49: 141–155.

[95] Miller DA, Nichols JD, McClintock BT, Grant EHC, Bailey LL, Weir LA. Improving occupancy estimation when two types of observational error occur: Non‐detection and species misidentification. Ecology. 2011; 92: 1422–1428. 21870616 10.1890/10-1396.1

[96] Morrison LW. Observer error in vegetation surveys: a review. J Plant Ecol. 2015; 9: 367–379.

[97] McCune B, Dey JP, Peck JE, Cassell D, Heiman K, Will-Wolf S, et al Repeatability of community data: species richness versus gradient scores in large-scale lichen studies. Bryologist. 1997; 100: 40–46.

[98] Gu W, Swihart RK. Absent or undetected? Effects of non-detection of species occurrence on wildlife–habitat models. Biol Conserv. 2004; 116: 195–203.

[99] Milberg P, Bergstedt J, Fridman J, Odell G, Westerberg L. Observer bias and random variation in vegetation monitoring data. J Veg Sci. 2008; 19: 633–644.

[100] Fitzpatrick MC, Preisser EL, Ellison AM, Elkinton JS. Observer bias and the detection of low‐density populations. Ecol Appl. 2009; 19: 1673–1679. 19831062 10.1890/09-0265.1

[101] Ahrends A, Rahbek C, Bulling MT, Burgess ND, Platts PJ, Lovett JC, et al Conservation and the botanist effect. Biol Conserv. 2011; 144: 131–140.

[102] Iknayan KJ, Tingley MW, Furnas BJ, Beissinger SR. Detecting diversity: emerging methods to estimate species diversity. Trends Ecol Evol. 2014; 29: 97–106. 10.1016/j.tree.2013.10.012 24315534 10.1016/j.tree.2013.10.012

[103] Robinson OJ, Ruiz‐Gutierrez V, Fink D. Correcting for bias in distribution modelling for rare species using citizen science data. Divers Distrib. 2018; 24: 460–472.

[104] McGill BJ, Etienne RS, Gray JS, Alonso D, Anderson MJ, Benecha HK, et al Species abundance distributions: moving beyond single prediction theories to integration within an ecological framework. Ecol Lett. 2007; 10: 995–1015. 10.1111/j.1461-0248.2007.01094.x 17845298 10.1111/j.1461-0248.2007.01094.x

[105] Danielsen F, Mendoza MM, Alviola P, Balete DS, Enghoff M, Poulsen MK, et al Biodiversity monitoring in developing countries: what are we trying to achieve? Oryx. 2003; 37:407–409.

[106] Rosenvald R, Lõhmus A, Kraut A, Remm L. Bird communities in hemiboreal old-growth forests: the roles of food supply, stand structure, and site type. For Ecol Manage. 2011; 262: 1541–1550.

[107] Isaac NJ, Mallet J, Mace GM. Taxonomic inflation: its influence on macroecology and conservation. Trends Ecol Evol. 2004; 19: 464–469. 10.1016/j.tree.2004.06.004 16701308 10.1016/j.tree.2004.06.004

[108] Lõhmus A. Collective analyses on “red-listed species” may have limited value for conservation ecology. Biodivers Conserv. 2015; 24: 3151–3153.

[109] Nichols JD, Hines JE, Sauer JR, Fallon FW, Fallon JE, Heglund PJ. A double-observer approach for estimating detection probability and abundance from point counts. Auk. 2000; 117: 393–408.

[110] Kendall WL, Peterjohn BG, Sauer JR. First-time observer effects in the North American breeding bird survey. Auk. 1996; 113: 823–829.

