# Supplementary material for: A simple survey protocol for assessing terrestrial biodiversity in a broad range of ecosystems
Source: PLoS One. 2018 Dec 12;13(12):e0208535. doi: 10.1371/journal.pone.0208535 (PMC6291155; doi:10.1371/journal.pone.0208535)

S1 Figure. Examples of contrasting forest systems surveyed using the fixed-area-fixed-effort approach and analysed on Figure 1. A1 – old *terra firma* forest in French Guiana. A2, B1 and B2 – eutrophic hemiboreal forest sites in Estonia (A2, old growth, B1 mature managed stand, B2 clear-cut with retention trees. C1, C2 Scots pine dominated forests in Estonia on drained peat and sandy soils, respectively.

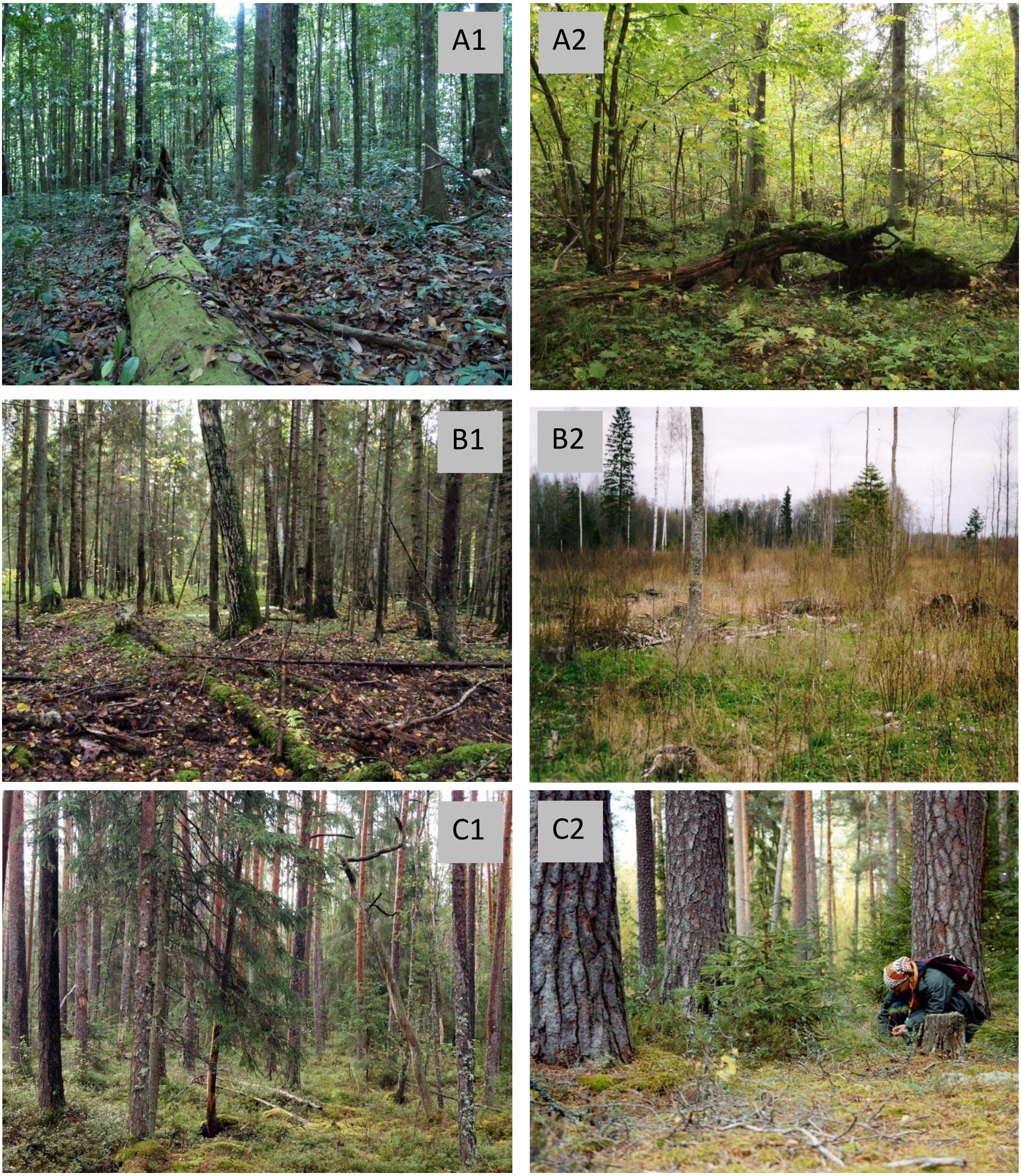

Supplement: S1 Fig — (PDF) [file pone.0208535.s001.pdf]
