# Supplementary material for: A simple survey protocol for assessing terrestrial biodiversity in a broad range of ecosystems
Source: PLoS One. 2018 Dec 12;13(12):e0208535. doi: 10.1371/journal.pone.0208535 (PMC6291155; doi:10.1371/journal.pone.0208535)
Supplement: S4 Table — (PDF) [file pone.0208535.s005.pdf]

S4 Table. Examples of different standard approaches proposed for plant and fungal monitoring schemes.

Taxon group	Eco-system	Measurement target	Basic method	Reference
Not specified	Diverse habitats, Amazonia	Multiple taxon groups	**Sampling units**: various-shaped plots (depending on terrain) in a fixed 1-km^2 grid over 25-km^2 areas. **Survey description**: not specified.	**Costa FR, Magnusson WE.** The need for large-scale, integrated studies of biodiversity – the experience of the program for biodiversity research in Brazilian Amazonia. Natureza & Conservação. 2010; 8: 3-12.
Ground vegetation (macroscopic photoautotrophs)	Diverse habitats	Vegetation composition and structure	**Sampling units:** nested rectangular replicate plots of varying sizes (min. 0.01 m^2) within 1000 m^2 basic plots. **Survey description:** exhaustive sampling; unlimited time. Record each species; also cover in subplots.	**Dengler J.** A flexible multi-scale approach for standardised recording of plant species richness patterns. Ecol Indic. 2009; 9:1169-1178.
Ground vegetation (vascular plants; lichens)	Diverse habitats,Africa	Vegetation composition and structure	**Sampling units:** nested rectangular replicate plots of 100, 1000, and 10 000 m^2 size. **Survey description:** exhaustive sampling; unlimited time. Record all species; in smaller plots, also include cover and abundance measurements.	**Jürgens N, Schmiedel U, Haarmeyer DH, Dengler J, Finckh M, Goetze D, Gröngröft A, Hahn K, Koulibaly A, Luther-Mosebach J, Muche G.** The BIOTA Biodiversity Observatories in Africa—a standardized framework for large-scale environmental monitoring. Environ Monit Assess. 2012; 184:655-78; **Zedda L, Gröngröft A, Schultz M, Petersen A, Mills A, Rambold G.** Distribution patterns of soil lichens across different biomes of southern Africa. J Arid Environ. 2011; 75:215–220.
Vascular plants, mosses	Diverse habitats, Switzer-land	Species diversity in different habitats, focus on common taxa	**Sampling unit:** circular 10-m^2 plot (radius 1.79 m). **Survey description:** exhaustive sampling; unlimited time limited. Record each species and its cover. Vascular plants sampled 1-2 times each year; mosses sampled in spring.	http://www.biodiversitymonitoring.ch/en/downloads.html
Vascular plants	Diverse habitats, Switzer-land	Species diversity in landscapes, focus on sparsely distributed taxa	**Sampling unit:** 2.5 km transect **Survey description:** Exhaustive survey twice a year; unlimited time. Record each species.	http://www.biodiversitymonitoring.ch/en/downloads.html
Vascular plants; bryophytes	Forest	Species list	**Sampling units**: systematically placed 1 ha plots. **Survey description**: time-limited surveys of species (1 h for vascular plants; 2 h for bryophytes).	**Nielsen SE, Haughland DL, Bayne E, Schieck J.** Capacity of large-scale, long-term biodiversity monitoring programmes to detect trends in species prevalence. Biodiv Cons. 2009; 18:2961-2978.

Vascular plants	Forest	Species list; woody species' abundance	**Sampling unit**: 50 x 50 m plot<br>**Survey description**: unlimited time. Record all species in the plot; simultaneous sampling by two persons encouraged. Systematically delineate five 5 m × 5 m subplots and count every shoot with a DBH > 1 cm.	**Borges PA, Cardoso P, Kreft H, Whittaker RJ, Fattorini S, Emerson BC, Gil A, Gillespie RG, Matthews TJ, Santos AM, Steinbauer MJ.** Global Island Monitoring Scheme (GIMS): a proposal for the long-term coordinated survey and monitoring of native island forest biota. Biodiv Cons. 2018; 7:1-20.
Bryophytes	Diverse habitats	Species richness and composition along environ-mental gradients	**Sampling unit:** plots within 1 ha localities; plot size habitat dependent (e.g., 100 x 100 cm in pastures, 5 x 5 cm in social areas, 30 x 30 cm in forests).<br>**Survey description:** not specified.	**Aranda SC, Gabriel R, Borges PA, De Azevedo EB, Lobo JM.** Designing a survey protocol to overcome the Wallacean shortfall: a working guide using bryophyte distribution data on Terceira Island (Azores). Bryologist. 2011; 114:611-24.
Arboreal bryophytes	Tropical forests	Species diversity	**Sampling unit:** three 2 x 2 m quadrats nested in 10 × 10 m plots; 3 trees in each quadrat<br>**Survey description:** on each tree, record all species in three 5 × 10 cm patches at three heights (0–50 cm, 0.5–1 m and 1–2 m).	**Ah-Peng C, Wilding N, Kluge J, Descamps-Julien B, Bardat J, Chuah-Petiot M, Strasberg D, Hedderson TA.** Bryophyte diversity and range size distribution along two altitudinal gradients: continent vs. island. Acta Oecol. 2012; 42: 58-65; **Gabriel R, Coelho MMC, Henriques DSG, Borges PAV, Elias RB, Kluge J, Ah-Peng C.** Long-term monitoring across elevational gradients to assess ecological hypothesis: a description of standardized sampling methods in oceanic islands and first results. Arquipélago. 2014; 31:45-67.
Lichens	Forests in Europe	Species diversity and indicator species on the tree bark	**Sampling unit:** 4-12 trees (not fixed), plot size not fixed (0.25 km^2- 1 km^2)<br>**Survey description:** on each tree exhaustive sampling in 4*5 sampling grid (each cell 10x10 cm). Unlimited time. Record each species and its frequency (no. of cells where present).	**Asta J, Erhardt W, Ferretti M, Fornasier F, Kirschbaum U, Nimis PL, Purvis OW, Pirintsos S, Scheidegger C, van Haluwyn C, Wirth V.** Mapping lichen diversity as an indicator of environmental quality. In: Nimis PL, Scheidegger C, Wolseley P, editors. Monitoring with Lichens- Monitoring Lichens. Kluwer Academic Publisher, Netherlands; 2002. pp. 273-279;<br>**Cristofolini F, Brunialti G, Giordani P, Nascimbene J, Cristofori A, Gottardini E, Frati L, Matos P, Batič F, Caporale S, Fornasier MF**. Towards the adoption of an international standard for biomonitoring with lichens—consistency of assessment performed by experts from six European countries. Ecol Indic. 2014; 45:63-7.
Lichens	Forests in USA (+	Species diversity	**Sampling unit:** 0.38-ha circular plot (radius 35 m)	**USDA Forest Service.** Forest Inventory and Analysis National Core Field Guide Volume I: Field Data Collection

	Canada, Mexico)		**Survey description:** time limited (up to 2h). Record each species on woody substrates >0.5 m from the ground and its abundance (based on the number of individual thalli; 4 categories from rare (1–3 individuals) to abundant (on more than half of all substrates).	Procedures for Phase 2 Plots, Version 7.0. Available at: http://www.fia.fs.fed.us/library/field-guides-methods-proc/docs/2015/Core-FIA-FG-7.pdf
Macrolichens	Parks	Species diversity	**Sampling unit:** 20-m^2 rectangular plot. **Survey description:** a method designed for citizen scientists, time limited (10 min). Take a photo of each corticolous macrolichen specimen encountered.	**Casanovas P, Lynch HJ, Fagan WF**. Using citizen science to estimate lichen diversity. Biol Cons. 2014; 171:1-8.
Conspicuous macrofungi	Diverse habitats	Species diversity	**Sampling unit:** large plots (size unspecified) including ten 100-m long transects; 20 circular 5-m^2 plots (radius 1.262 m) per transect. **Survey description:** Collect all species. Resampling every 2 weeks during the fruiting season.	**Mueller GM, Schmit JP, Hubndorf SM, Ryvarden L, O'Dell TE, Lodge DJ, Leacock PR, Mata M, Umania L, Czederpiltz DL.** Recommended protocols for sampling macrofungi. In: Mueller GM, editor. Biodiversity of fungi: inventory and monitoring methods. Elsevier Academic Press, Boston; 2004. pp. 168-172.
Fungi inhabiting large woody substrates	Forest	Species diversity	**Sampling unit:** 30 logs (>20 cm diam and >2 m in length) in each decay class in a plot (size unspecified) **Survey description:** Collect 2-3 sporocarps of each common species and all sporocarps of rare species. Resampling every 2 weeks during the fruiting season.	**Mueller GM, Schmit JP, Hubndorf SM, Ryvarden L, O'Dell TE, Lodge DJ, Leacock PR, Mata M, Umania L, Czederpiltz DL.** Recommended protocols for sampling macrofungi. In: Mueller GM, editor. Biodiversity of fungi: inventory and monitoring methods. Elsevier Academic Press, Boston; 2004. pp. 168-172.
